# Comparative efficacy and safety of pharmacologic, laser, and combination therapies for infantile hemangioma: a systematic review and network meta-analysis with evidence certainty assessment

**DOI:** 10.3389/fphar.2026.1882566

**Published:** 2026-09-09

**Authors:** Mingxuan Kong, Shijie Zhang, Dan Ren, Jian Chang

**Affiliations:** Department of Pediatric Oncology, Children’s Medical Center, the First Hospital of Jilin University, Changchun, China

**Keywords:** combination therapies, efficacy and safety, infantile hemangioma, network meta-analysis, β-blockers

## Abstract

**Introduction:**

Infantile hemangiomas (IH) are the most common childhood vascular tumors, potentially complicated by ulceration and permanent functional or aesthetic sequelae. Current treatments include corticosteroids, β-blockers, and lasers, among others, existing evidence relies predominantly on direct pairwise comparisons. Consequently, the relative benefits and risks across these modalities remain inadequately defined. We conducted a systematic review and network meta-analysis (NMA) evaluating pharmacologic, laser, and combination therapies for IH to establish treatment hierarchies and inform clinical practice and future guidelines.

**Methods:**

Following a PROSPERO-registered protocol (CRD420261388356), we searched PubMed, Embase, the Cochrane Library, and Web of Science from inception to 1 March 2026, for randomized controlled trials comparing various interventions for IH. The primary endpoint was excellent response rate. Secondary outcomes included other response rates, VAS/HAS changes, healing time, treatment emergent adverse events, and recurrence. After independent risk-of-bias assessment (RoB2) and data extraction, we performed a random-effects NMA. Treatments were ranked using SUCRA probabilities, and evidence certainty was appraised via GRADE and CINeMA.

**Results:**

The synthesis comprised 25 trials (2044 patients) evaluating 16 distinct interventions. Risk of bias was low in six trials, raised “some concerns” in 14, and high in 5. Overall, the evidence body was of moderate certainty. Regarding the primary endpoint, intralesional bleomycin combined with oral propranolol yielded significantly higher excellent response rates than oral propranolol alone (RR = 1.55, 95% CI 1.28–1.89; high certainty). Topical timolol with intralesional lauromacrogol (RR = 7.98, 95% CI 1.52–41.79) and oral propranolol with corticosteroids (RR = 2.00, 95% CI 1.03–3.88) showed higher excellent response rates, supported by moderate-certainty evidence. No statistically significant differences in adverse-event incidence were detected across evaluated regimens, but the safety data were limited. Oral atenolol was associated with numerically lower recurrence rates compared to oral propranolol, but the difference did not reach statistical significance (RR = 0.64, 95% CI 0.32–1.28; moderate certainty).

**Discussion:**

Oral propranolol remains the standard of care for IH. However, our analysis shows that some combination regimens showed favorable efficacy for excellent clinical response. Still, sparse comparative data and low-certainty evidence currently obscure the optimal combination strategy. Defining precise risk-benefit profiles requires rigorous head-to-head trials utilizing uniform outcome measures.

**Systematic Review Registeration:**

https://www.crd.york.ac.uk/PROSPERO/view/CRD420261388356; Identifier: CRD420261388356.

## 1 Introduction

Infantile hemangiomas (IHs) are the most common pediatric vascular tumors, affecting approximately 4.5% of infants. They typically undergo rapid proliferation during the first 8 weeks of life (Tollefson and Frieden, 2012), followed by growth arrest in most patients by 5 months of age (Chang et al., 2008). Based on the depth of tissue involvement, these lesions are morphologically classified into superficial, deep, and mixed subtypes. While the underlying pathogenesis is complex, prior meta-analyses have established female sex, low birth weight, prematurity, and maternal prenatal medication exposure as primary risk factors (Sun et al., 2024; Ding et al., 2020). Although the majority of IHs spontaneously involute, nearly 10% of cases develop severe or potentially life-threatening complications, including ulceration, functional impairment, and structural disfigurement (Cheng and Friedlander, 2016; Satterfield and Chambers, 2019; Darrow et al., 2015; Krowchuk et al., 2019). Beyond direct physical morbidity, these complicated lesions exert a substantial psychosocial toll on patients and caregivers, frequently impairing overall quality of life and disrupting normal childhood development (Pensabene et al., 2022; Zweegers and van der Vleuten, 2012).

The clinical management of IHs currently involves multiple interventional strategies, such as beta-blockers (e.g., propranolol, atenolol, nadolol, timolol), corticosteroids, and laser therapies (Sebaratnam et al., 2021). Although oral propranolol is the established first-line therapy, its clinical utility is frequently limited by poor adherence driven by potential systemic adverse events, including sleep disturbances, lethargy, irritability, bradycardia, and hypoglycemia (Langley and Pope, 2015). Given these risks, some caregivers opt for watchful waiting over active intervention. Alternative oral beta-blockers, such as atenolol and nadolol, demonstrate preliminary efficacy, however, the lack of robust randomized controlled trials (RCTs) prevents definitive comparison with propranolol (Calderón et al., 2020; Ballona et al., 2020; Fernandez et al., 2025). Topical timolol is widely utilized for its favorable tolerability, though transdermal systemic absorption remains a clinical consideration (McMahon et al., 2012). Due to well-characterized toxicity, systemic corticosteroids are now largely restricted to second-line or combination roles for refractory cases (Price et al., 2011; Aly et al., 2015; George et al., 2004), while laser therapy serves primarily as an adjunct for residual or treatment-resistant lesions. Surgical excision also remains an important selective option in the current therapeutic algorithm, particularly for residual fibrofatty tissue or contour deformity after involution, lesions refractory to or unsuitable for medical therapy, selected ulcerated or bleeding lesions amenable to resection, or cases requiring functional or cosmetic reconstruction (Krowchuk et al., 2019). Despite the availability of these varied treatments, high-certainty head-to-head evidence is scarce. The existing literature is further complicated by substantial methodological heterogeneity, particularly regarding unstandardized clinical endpoints. As a result, the comparative safety and efficacy of these interventions remain unclear, necessitating rigorous, standardized analyses to accurately guide clinical decision-making.

Although prior systematic reviews have evaluated IH treatments, their clinical applicability is often hindered by methodological constraints. Most existing reviews either restrict their scope to single therapeutic classes (Huang et al., 2024; Huang et al., 2022) or rely excessively on probabilistic ranking metrics (e.g., SUCRA) without adequately appraising evidence certainty or absolute effect differences (Yang et al., 2020). Network meta-analysis (NMA) addresses these gaps by integrating direct and indirect evidence, allowing for the simultaneous comparison of multiple interventions. To avoid the pitfalls of isolated rankings and ensure our findings are clinically meaningful, this NMA incorporates certainty-of-evidence frameworks (GRADE and CINeMA) alongside minimal clinically important difference (MCID) thresholds. By contextualizing statistical significance with clinical relevance, this study aims to provide robust, evidence-based guidance to support shared decision-making in IH management.

## 2 Methods

### 2.1 Materials and methods

This network meta-analysis (NMA) was conducted in accordance with the PRISMA Extension for Network Meta-Analyses (PRISMA-NMA) (Hutton et al., 2015) (Supplementary Table S1). The study protocol was prospectively registered in PROSPERO (CRD420261388356).

### 2.2 Data sources and search strategy

We systematically searched PubMed, EMBASE, the Cochrane Library, and Web of Science from database inception through 1 March 2026, without language restrictions. The search strategy integrated controlled vocabulary (e.g., MeSH, Emtree) and free-text terms. Key search queries included “infantile hemangioma,” “Adrenergic beta-Antagonists,” “Propranolol,” “Atenolol,” “Nadolol,” “Timolol,” “Carteolol,” “Glucocorticoids,” “Captopril,” “Vincristine,” “Sirolimus,” “Interferon,” “Lasers,” “Bleomycin,” “Polidocanol,” “surgery,” “Sclerotherapy,” and “randomized clinical trial”. (Supplementary Table S2).

### 2.3 Selection criteria

Studies were included if they met the following criteria: (1) randomized controlled trials (RCTs) evaluating treatments for infantile hemangioma; (2) comparison of at least two therapeutic regimens; and (3) reporting of one or more prespecified clinical outcomes. These outcomes included excellent response (>75% reduction in tumor size), marked response (>50% reduction), total response (>25% reduction), Visual Analogue Scale (VAS) metrics (standardized to a 0–10 scale, higher scores indicate better clinical outcome), Hemangioma Activity Score (HAS) (lower scores indicate better clinical outcomes), healing time, treatment emergent adverse event, or recurrence. Exclusion criteria encompassed non-randomized study designs (e.g., reviews, case reports), intra-patient split-lesion trials, and studies with ambiguous or undefined efficacy parameters. Two investigators independently screened the titles and abstracts of retrieved records, advancing potentially eligible articles to full-text review. To preclude duplicate patient counting from overlapping cohorts, the reviewers cross-checked trial data, retaining only the most recent and comprehensive publication for any given dataset.

### 2.4 Screening process and data extraction

Two researchers independently carried out the data extraction process. Any disagreements between them were settled through mutual discussion, or when necessary, by consulting a third adjudicator. The gathered information encompassed trial metadata (e.g., primary author, year of publication, and geographical setting), baseline demographic profiles (e.g., participant age, gender distribution, and cohort size), as well as detailed intervention metrics (e.g., specific regimens and length of treatment).

Treatment nodes were prespecified according to the active agent, route of administration, and treatment strategy. The monotherapy or single-treatment nodes included oral propranolol, oral atenolol, oral nadolol, oral prednisolone, topical timolol, topical propranolol, laser therapy, intralesional propranolol, and watchful waiting. Combination regimens were defined as separate nodes, including oral propranolol with laser therapy, oral propranolol with topical timolol, oral propranolol with oral corticosteroid, oral propranolol with intralesional lauromacrogol, laser therapy with topical propranolol, topical timolol with intralesional lauromacrogol, and oral propranolol with intralesional bleomycin. Interventions with different routes of administration, mechanisms, or treatment strategies were not merged. For example, oral, topical, and intralesional propranolol were treated as separate nodes because they differ in systemic exposure, local drug concentration, treatment intensity, and clinical indication. Combination regimens were also treated as distinct nodes because their effects reflect the combined treatment strategy rather than either component alone. Interventions within the same node were considered clinically comparable because they shared the same active agent or therapeutic modality, route of administration, and overall treatment strategy.

Because the original definitions of treatment response varied across trials, we extracted the original response grading criteria from each eligible study and matched the reported categories to our prespecified thresholds. Outcome data were included in a given analysis only when the reported response category corresponded to the predefined threshold for that endpoint. Specifically, excellent response was defined as >75% reduction in tumor size, marked response as >50% reduction, and total response as >25% reduction. If a study reported response categories that did not match the predefined threshold for a specific endpoint, those data were not included in the corresponding analysis.

Regarding continuous endpoints, we captured the mean variations from baseline alongside their corresponding standard deviations (SDs). In instances where studies provided only pre-treatment and post-treatment values, the mean change was computed by determining the mathematical difference between the two time points. To estimate the standard deviation for this change-score (SDchange), we utilized the SDs from the baseline (SDbaseline) and the final assessment (SDendpoint), factoring in a correlation coefficient (R) *via* the following established equation:

$${SD}_{change}=\sqrt{\text{Baseline }{\text{SD}}^{2}+\text{Endpoint }{\text{SD}}^{2}-2R\cdot \text{Baseline SD}\cdot \text{Endpoint SD}}$$

In cases where the correlation coefficient could not be empirically derived from the included literature, a primary correlation coefficient (R) of 0.5 was adopted for missing variance imputation; alternative values of 0.25 and 0.75 were subsequently employed in sensitivity analyses to account for parameter uncertainty (Supplementary Figure S13). Finally, for dichotomous variables, we tallied the number of observed events and the total number of subjects assigned to each respective arm.

### 2.5 Quality assessment

We assessed the risk of bias in the included trials using the revised Cochrane risk-of-bias tool (RoB 2). Evaluations covered the five standard domains: the randomization process, deviations from intended interventions, missing outcome data, measurement of the outcome, and selection of the reported results. Both domain-specific and overall risk of bias were classified as ‘low risk’, ‘some concerns’, or ‘high risk’ (Sterne et al., 2019).

### 2.6 Statistical analysis

Statistical analyses were conducted using Stata 18.0 MP. We calculated mean differences (MDs) for continuous variables measured on the same scale, and risk ratios (RRs) for binary outcomes; all effect estimates are reported with 95% confidence intervals (CIs). For multi-arm trials, we adjusted for within-study correlations to avoid double-counting shared control groups. A standard 0.5 continuity correction was applied to arms with zero events. Before conducting the network meta-analysis, we assessed the plausibility of the transitivity assumption by comparing key trial-level effect modifiers across treatment comparisons, including treatment indication, lesion subtype and depth, anatomical location, disease stage, baseline lesion burden, baseline severity scale or activity score, high-risk features or complications, previous infantile hemangioma treatment, treatment duration, and follow-up duration. Assuming transitivity, we performed a random-effects network meta-analysis (NMA). Between-study heterogeneity (τ^2^) was estimated using restricted maximum likelihood (REML). We evaluated global inconsistency in closed loops using the design-by-treatment interaction model, and assessed local inconsistency *via* the node-splitting method (P < 0.05 indicated significant disagreement). Loop-specific inconsistency factors (IFs) were also calculated; 95% CIs including zero indicated concordance between direct and indirect evidence. Network geometry was visualized using standard network plots, with node size and edge thickness proportional to the total sample size and the number of direct comparisons, respectively. Treatments were ranked using the surface under the cumulative ranking curve (SUCRA), mean ranks, and the probability of being the best treatment. When ten or more trials were available for a given comparison, comparison-adjusted funnel plots were generated to screen for small-study effects and publication bias. Finally, we tested the robustness of our findings with a leave-one-out sensitivity analysis and explored potential effect modifiers *via* univariable network meta-regression (reporting regression coefficients, 95% CIs, and Wald test P-values).

### 2.7 GRADE grading

The credibility of the pooled network estimates was appraised applying the Confidence in Network Meta-Analysis (CINeMA) approach. Given that our analysis was restricted strictly to randomized controlled trials (RCTs), the initial quality rating for all comparisons began at ‘high’. The evaluation encompassed the six established CINeMA criteria. To determine within-study bias, Risk of Bias 2 (RoB 2.0) evaluations were merged with the contribution matrix to yield a weighted risk profile per effect estimate. Transitivity—and thus indirectness—was verified by cross-examining predefined clinical modifiers across studies, such as baseline severity, intervention intensity, and length of follow-up. Imprecision was quantified by referencing a predetermined minimal important difference (MID). Concurrently, heterogeneity was analyzed by juxtaposing prediction intervals against this MID, complemented by the between-trial variance parameter (τ^2^). For closed-loop configurations, incoherence was scrutinized employing both the design-by-treatment interaction approach and the SIDE (node-splitting) functionality inherent to the CINeMA software. Lastly, reporting bias was screened by investigating gray literature, checking clinical trial registries, and visually assessing comparison-adjusted funnel plots. Adhering to the GRADE methodology, evidence quality was penalized by one or two tiers in the presence of “some” or “major” concerns, respectively. This process resulted in an ultimate classification of high, moderate, low, or very low confidence.

## 3 Results

### 3.1 Characteristics of included trials

**FIGURE 1:**
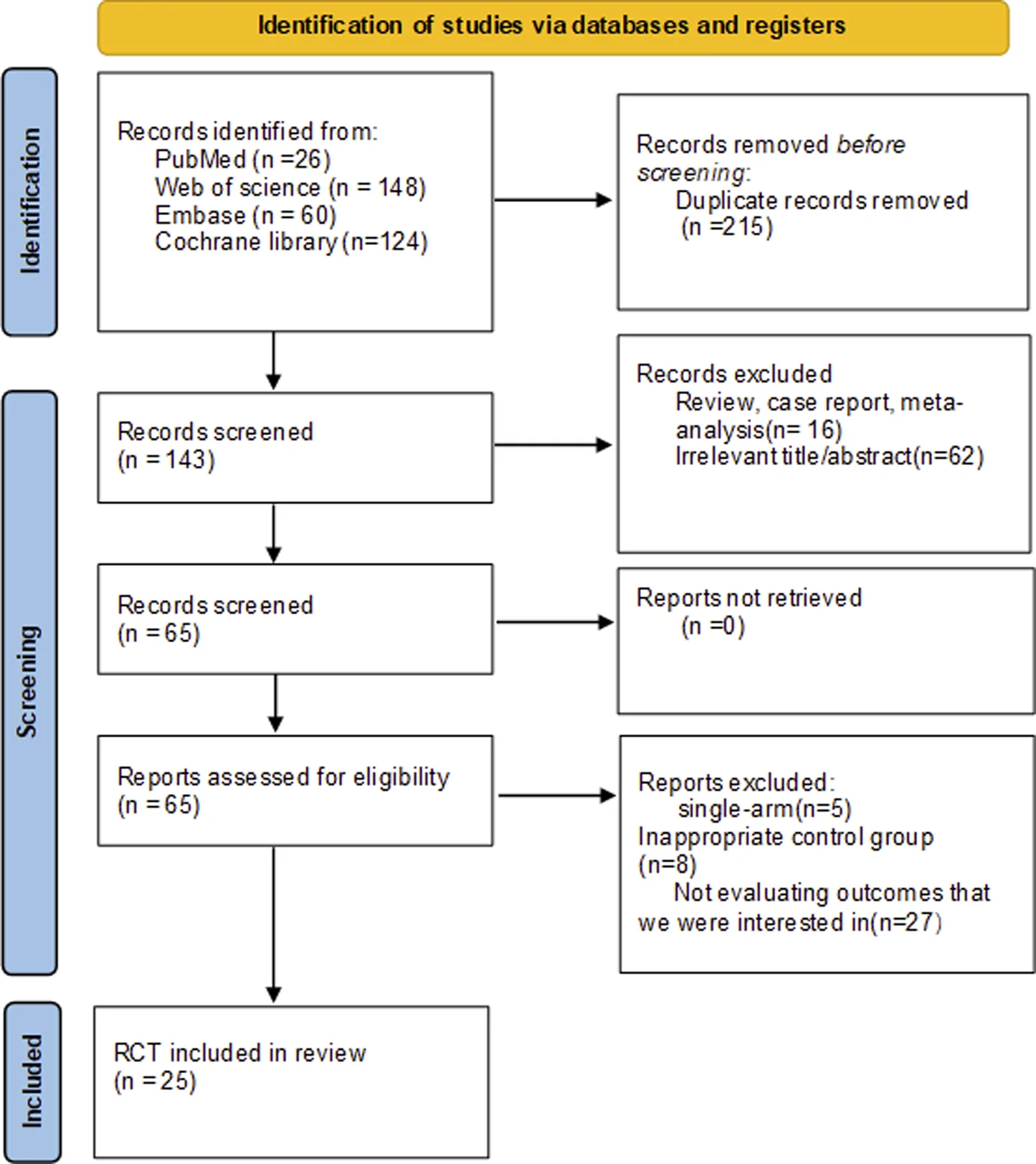
PRISMA flow diagram of study selection.

Our initial search yielded 358 records. After deduplication and title/abstract screening, we reviewed 65 full-text articles, ultimately including 25 studies (Figure 1). The final network comprised a pooled cohort of 2,044 patients evaluated across 16 treatment arms. These interventions included monotherapies—oral propranolol (OP), oral atenolol (OA), oral nadolol (ON), oral prednisolone (OCS), topical timolol (TT), topical propranolol (TP), laser therapy (Laser), intralesional propranolol (ILP), and watchful waiting (WW)—as well as seven combination regimens: OP with Laser (OP-Laser), OP with TT (OP-TT), OP with OCS (OP-OCS), OP with intralesional lauromacrogol (OP-ILL), Laser with TP (Laser-TP), TT with ILL (TT-ILL), and OP with intralesional bleomycin (OP-ILB). The included studies encompassed multiple global data centers, involving research from China, Egypt, the USA, and various other countries. The overall sample size was moderate, consisting primarily of female infants (mostly with a mean age of <6 months). Comprehensive study-level baseline characteristics and key clinical effect modifiers for all included trials are detailed across Table 1 and Supplementary Table S32. Overall, the patient populations and clinical presentations were reasonably balanced across the treatment nodes. The majority of studies evaluated infants with hemangiomas in the actively proliferative phase, with a comparable distribution of anatomical locations (predominantly head and neck). Follow-up durations generally aligned across the major systemic and topical treatment nodes (typically spanning 6 months), supporting clinical comparability and the plausibility of the transitivity assumption. Furthermore, the original response grading criteria and percentage reduction thresholds reported in the included trials are summarized in Supplementary Table S31 The definitions, dose ranges and treatment parameters of the treatment nodes used in the network meta-analysis are summarized in Supplementary Table S33.

**TABLE 1:** Characteristics of the included studies.

First author	Year	Mean age (months)	Sex (M:F)	Sample	Intervention time (month)	Experimental intervention	Control intervention	Clinical classification	Location	Country
Gong et al. (2015)	2016	5.5	15:24	39	6	0.5% timolol twice daily + oral propranolol 1.0 mg/kg/d	0.5% timolol twice daily; oral propranolol 1.5 mg/kg/d	Superficial	Head	China
Ji et al. (2021)	2021	2.35	90:287	377	6	Oral atenolol 0.5 mg/kg/d for 1 week and increased to 1.0 mg/kg/d in week 2	Oral propranolol 1 mg/kg/d for 1 week and increased to 2.0 mg/kg/d in week 2	Superficial 48 Mixed 329	Head 199 trunk + extremity 178	China
Ashraf et al. (2023)	2023	4.05	17:23	40	9	Oral atenolol 1 mg/kg/d	Oral propranolol 2.0 mg/kg/d	Superficial 23 Mixed 16 Deep 1	Head 25 Trunk 11 Limb 4	India
Zhao et al. (2013)	2013	7.5	40:101	141	6	0.5% timolol once daily	Oral propranolol 1.0 mg/kg/d	Superficial	Head 105 Trunk 29 Lim b7	China
Li et al. (2016)	2016	6.5	13:18	31	8	0.5% timolol twice daily + oral propranolol 1.0 mg/kg/d	Oral propranolol 1.5 mg/kg/d	Mix	Head	China
Marey et al. (2018)	2018	5.15	12:13	25	8	0.5% timolol twice daily + oral propranolol 1.0 mg/kg/d for 2 weeks and increased to 2 mg/kg/d for week 3	Oral propranolol 1 mg/kg/d for 2 weeks and increased to 2.0 mg/kg/d in week 3	Superficial	Head	Egypt
Ehsani et al. (2014)	2014	8.25	3:16	19	4	PDL (spot size 7 mm, 12 J/cm^2^, pulse duration 1.5 m, dynamic cooling device 40/40), 3 sessions, once every 4 weeks, with 50% over lapped shuts	The same PDL sessions with topical propranolol 1%, applied twice a day	Superficial and mix	Head 10 Trunk 4 Limb 5	Iran
Liu et al. (2017)	2017	3.18	38:72	110	till lesions disappeared	PDL (spot size 7 mm, 7–11 J/cm^2^, pulse duration 2 m), 4–6 sessions, once every 4 weeks, with 10%–20% over lapped shuts	The same PDL sessions with oral propranolol 2 mg/kg/d	Superficial	NR	China
Sun et al. (2018)	2018	3.5	42:58	100	till lesions disappeared	Long-pulse 1,064 nm Nd:YAG laser (spot size 9 mm, pulse width 12 msec, 40–70 J/cm^2^), every 3 weeks, with <10% over lapped + oral propranolol 2 mg/kg/d	Oral propranolol 2 mg/kg/d	Superficia l40 Mixed 60	NR	China
Yang et al. (2018a)	2018	3.5	46:50	96	6	PDL (spot size 7 mm, 8–15 J/cm^2^, pulse duration 0.45–10 m, dynamic cooling device 40/40), every 2–4 weeks + topical propranolol	Topical propranolol	Superficial	Head 58 trunk 21 limb 17	China
Zaher et al. (2013)	2013	8.82	6:39	45	9	Oral propranolol 2.0 mg/kg/d	Topical propranolol 1%, twice daily; intralesional propranolol (0.2 mL injected per 1 cm of lesion diameter with a maximum volume of 1 mL)	NR	Head 33 Trunk 9 Limb 3	Egypt
Tawfik and Alsharnoubi (2015)	2015	11.45	15:45	60	6	0.5% timolol twice daily	Superficial: PDL (spot size 7 mm, 4.5–6 J/cm^2^, pulse duration 6 m), 1s delay, Nd:YAG Laser (pulse duration 6 m, 30–40 J/cm^2^) Mixed: PDL (spot size 7 mm, 6–7.5 J/cm^2^, pulse duration 10 m), 1s delay, Nd:YAG Laser (pulse duration 15 m, 30–40 J/cm^2^) every month for a maximum of six sessions.	Superficia l50 Mixed 10	Head3 7 Trunk 16 Limb 7	Egypt
Bauman et al. (2014)	2014	3.25	5:14	19	4	Oral prednisolone 2.0 mg/kg/d	Oral propranolol 2.0 mg/kg/d	Superficial 5 Mixed 10 Deep 4	Head 17 Trunk 1	America
Kim et al. (2017)	2017	3.3	15:19	34	4	Oral prednisolone 2.0 mg/kg/d	Oral propranolol 2.0 mg/kg/d	NR	Head 26 Trunk 4 Limb 6	Korea
Malik et al. (2013)	2013	4.9	12:18	30	6	Oral prednisolone 1.0 mg/kg/d + propranolol 1–3 mg/kg/d; oral prednisolone 1.0 mg/kg/d	Oral propranolol 1–3 mg/kg/d	Superficia l16 Mixed 8 Deep 6	Head 20 other	India
Tan et al. (2012)	2012	2.5	28:69	97	6	Nd:YAG Laser (pulse duration 20–50 m, 170–240 J/cm^2^cm^2^) every 6 weeks + oral propranolol 0.5 mg/kg/d for 2 weeks, increased to 0.8 mg/kg/d in week 3, increased to 1 mg/kg/d in week 5	Nd:YAG Laser (pulse duration 20–50 m, 170–240 J/cm^2^) every 6 weeks; oral propranolol 0.5 mg/kg/d for 2 weeks, increased to 0.8 mg/kg/d in week 3, increased to 1 mg/kg/d in week 5	NR	NR	China
Abdel Wahab et al. (2017)	2017	5.37	19:29	48	9	Topical propranolol 3 times a day	Oral propranolol 1–3 mg/kg/d	Superficial	Head 28 Trunk 11 Limb 9	Egypt
Pope et al. (2022)	2022	3.15	40:57	71	6	Oral nadolol 2 mg/kg/d	Oral propranolol 2 mg/kg/d	Superficial 2 Mixed 58 Deep 11	head	Canada
Cheng et al. (2020)	2020	2.78	14:27	41	6	0.5% timolol	Watchful waiting	Superficial	Head 25 Trunk 14 Limb 2	China
Mehta et al. (2019)	2019	13.3	11:9	20	6	Intralesional injection of propranolol hydrochloride in a dose of 0.2 mL per cm of the LLD with a maximum dose of 1 mL, 3 sessions, once every 4 weeks	Oral propranolol 3 mg/kg/d	NR	head	India
Aly et al. (2015)	2015	4.59	8:32	40	6	Oral prednisolone 2.0 mg/kg/d + propranolol 2 mg/kg/d	Oral propranolol 2 mg/kg/d	Superficia l15 Mixed 25	Head 35 Trunk 1 Limb 4	Egypt
Guo et al. (2025)	2025	5.33	122:138	260	6	Oral propranolol 1.0 mg/kg/d + intralesional injection of bleomycin	Oral propranolol 1.0 mg/kg/d	NR	Head 160 Trunk 67 Limb 33	China
Ma et al. (2024)	2024	3.6	17:50	67	6	Oral propranolol 2.0 mg/kg/d + intralesional injection of lauromacrogol for 2–4 times within 6 months	Oral propranolol 2.0 mg/kg/d	Superficial 3 Mixed 22 Deep 42	Head 25 Trunk 25 Limb 14 Mixed 3	China
Zhang et al. (2022)	2022	5.3	66:84	150	6	0.5% timolol twice daily + intralesional injection of lauromacrogol for 1–4 times	0.5% timolol twice daily	Superficial 36 Mixed 82 Deep 32	Head 98 Trunk 35 Limb 17	China
Kashif et al. (2018)	2018	3.46	49:35	84	6	Oral prednisolone 2.0 mg/kg/d	Oral propranolol 3.0 mg/kg/d	NR	Head 64 Trunk 14 Limb 21	Pakistan

### 3.2 ROB2 literature quality assessment

**FIGURE 2:**
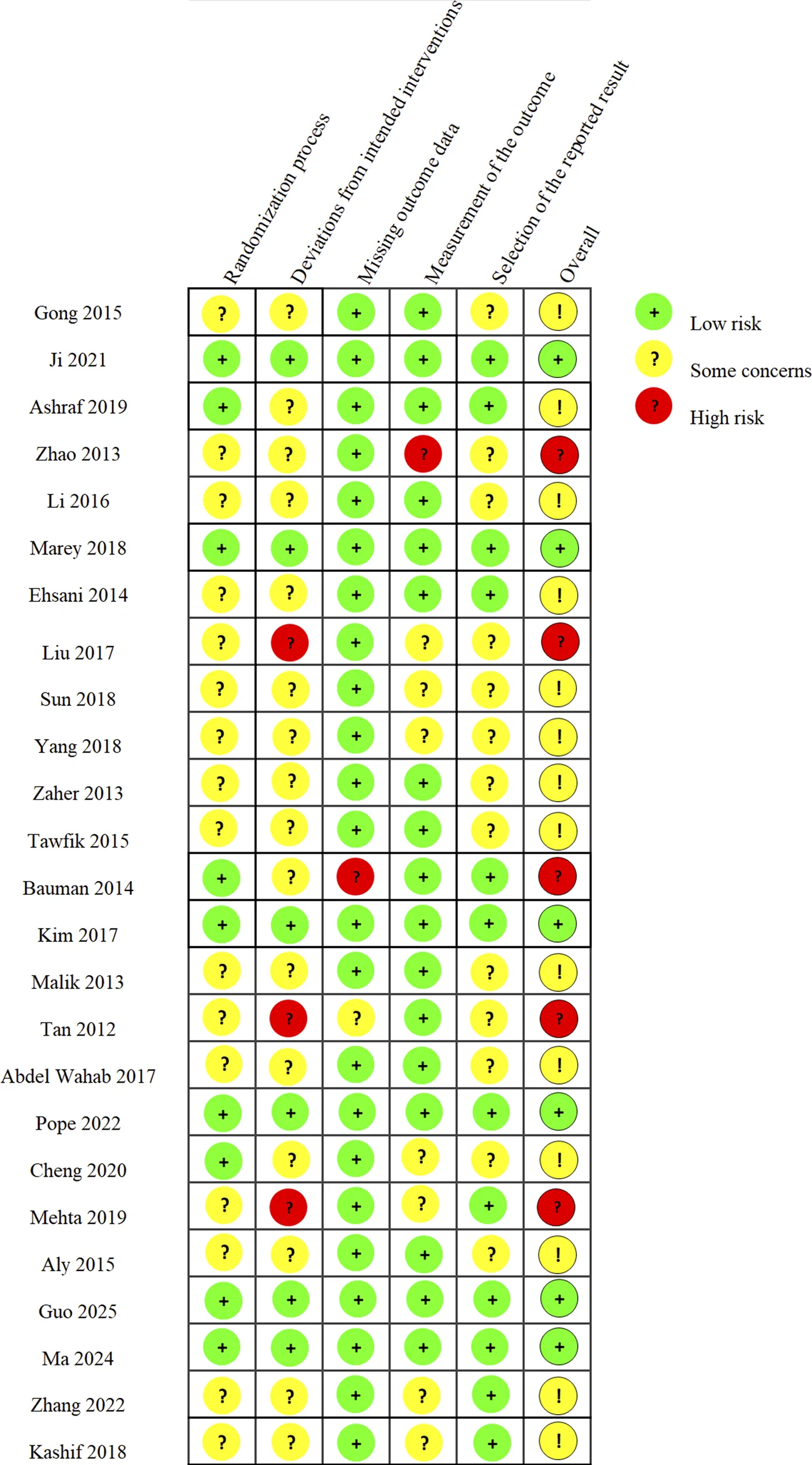
ROB2 risk of bias assessment.

Risk of bias for the 25 trials was evaluated using the Cochrane RoB 2 tool (Figure 2). Overall, six trials (24%) were rated as low risk, 14 (56%) had some concerns, and 5 (20%) were at high risk. Domain-level assessments revealed that while studies generally performed well regarding missing outcome data (n = 23 at low risk) and outcome measurement (n = 17), methodological weaknesses were heavily concentrated in deviations from intended interventions (n = 6) and the randomization process (n = 9). 12 trials were at low risk for the selection of the reported result.

### 3.3 Consistency and heterogeneity

Networks for marked effective rate and recurrence rate contained closed loops. Global inconsistency models (P > 0.05), local node-splitting (P > 0.05 for all nodes; Supplementary Tables S4 and S5), and loop-specific inconsistency factors (95% CIs crossing zero; Supplementary Tables S6 and S7) demonstrated robust agreement between direct and indirect evidence. Consequently, primary analyses for these outcomes proceeded using consistency models.

Conversely, networks for excellent response rate, total cure rate, Visual Analogue Scale (VAS), Hemangioma Activity Score (HAS), healing time and treatment emerge adverse event rate (TEAE) lacked closed loops, precluding formal incoherence testing. We synthesized these outcomes under the consistency assumption, supported by high clinical comparability across trial baselines (Table 1), with only minor variations noted in age and follow-up duration. However, to appropriately account for the unmeasurable incoherence, we downgraded the evidence certainty for these specific networks for indirectness.

Between-study heterogeneity was mild for marked response rate (τ = 0.12) and TEAE (τ = 0.25), but negligible for both excellent response rate and total response rate (τ < 0.001).

### 3.4 Results of network meta-analysis with different outcomes

#### 3.4.1 Efficacy

##### 3.4.1.1 Core efficacy endpoints

###### 3.4.1.1.1 Excellent response rate

**FIGURE 3:**
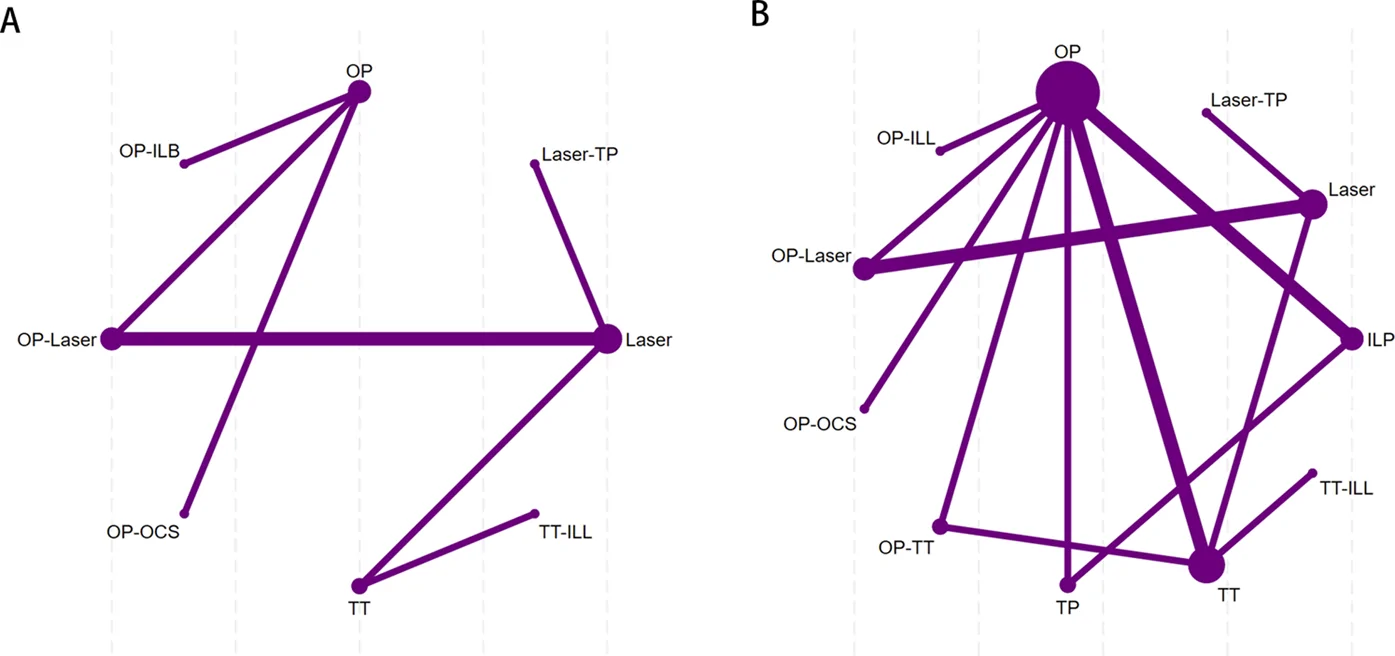
Network maps showing connections between pharmacologic, laser, and combination therapies for IH: **(A)** Excellent Response Rate **(B)** Marked Response Rates.

**FIGURE 4:**
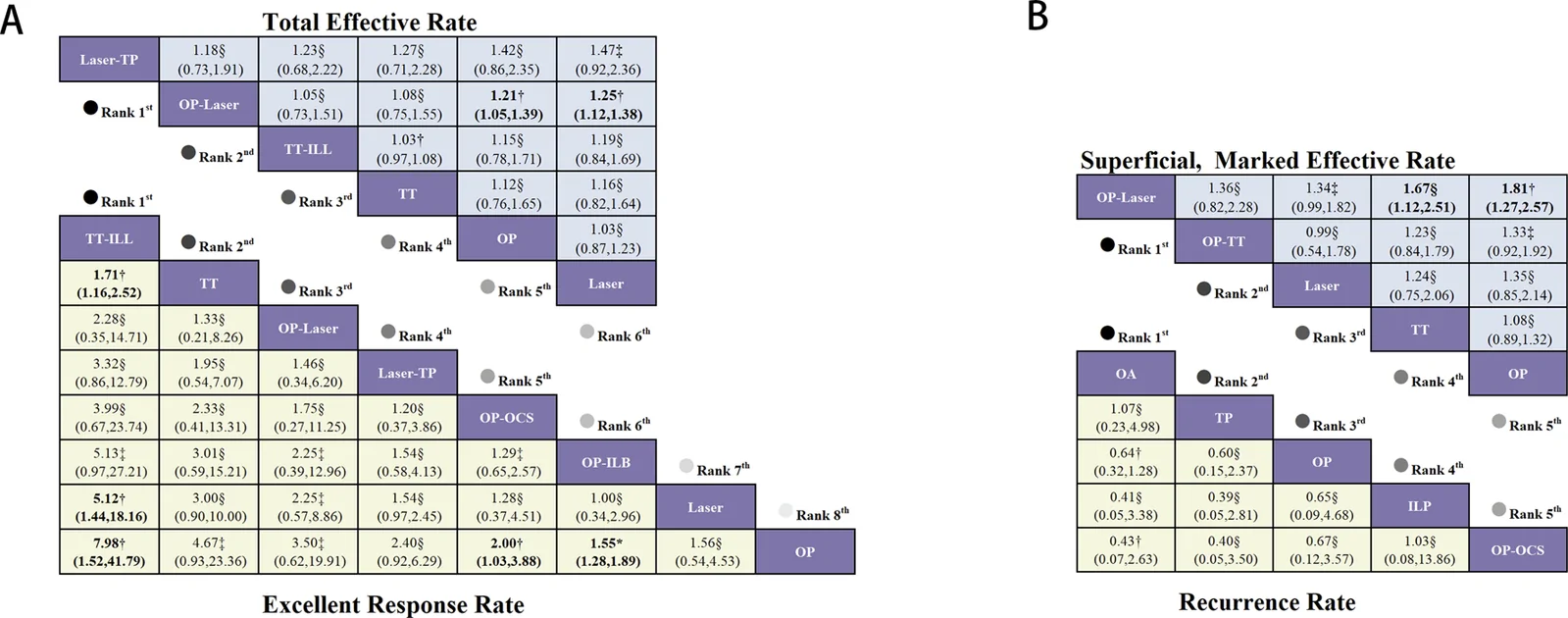
League matrix evaluating pharmacological, laser, and combined interventions for IH. **(A)** Comparative estimates for the Excellent Response Rate are detailed in the bottom-left section (highlighted in yellow), whereas the outcomes for the Total Effective Rate are displayed in the top-right section (shaded in blue). **(B)** The relative effects concerning the Recurrence Rate occupy the lower-left area (yellow shading), with the upper-right area (blue shading) illustrating the Marked Effective Rate specifically for superficial IH. Based on the GRADE framework, evidence certainty levels are marked as: * high, † moderate, ‡ low, and § very low.

Eight studies (835 infants) evaluated excellent response rates across eight intervention arms (Figure 3A). Compared to OP alone, higher excellent response rates were observed for OP-ILB (RR = 1.55, 95% CI 1.28–1.89; high-certainty evidence), OP-OCS (RR = 2.00, 95% CI 1.03–3.88; moderate certainty), and TT-ILL (RR = 7.98, 95% CI 1.52–41.79; moderate certainty) (Figure 4A). In the ranking analysis, TT-ILL had the highest SUCRA value (SUCRA = 95.2%; PrBest = 76.9%; mean rank = 1.3), followed by TT based on SUCRA (73.5%) and mean rank (2.9), rankogram analysis indicated that Laser-TP had a substantially higher probability of being the absolute best treatment compared to TT alone (18.1% vs. 0.2%) (Supplementary Figure S3). However, these ranking results should be interpreted together with the corresponding effect estimates, confidence intervals, and certainty of evidence, particularly because the estimate for TT-ILL was imprecise.

###### 3.4.1.1.2 Marked and total response rates

**FIGURE 5:**
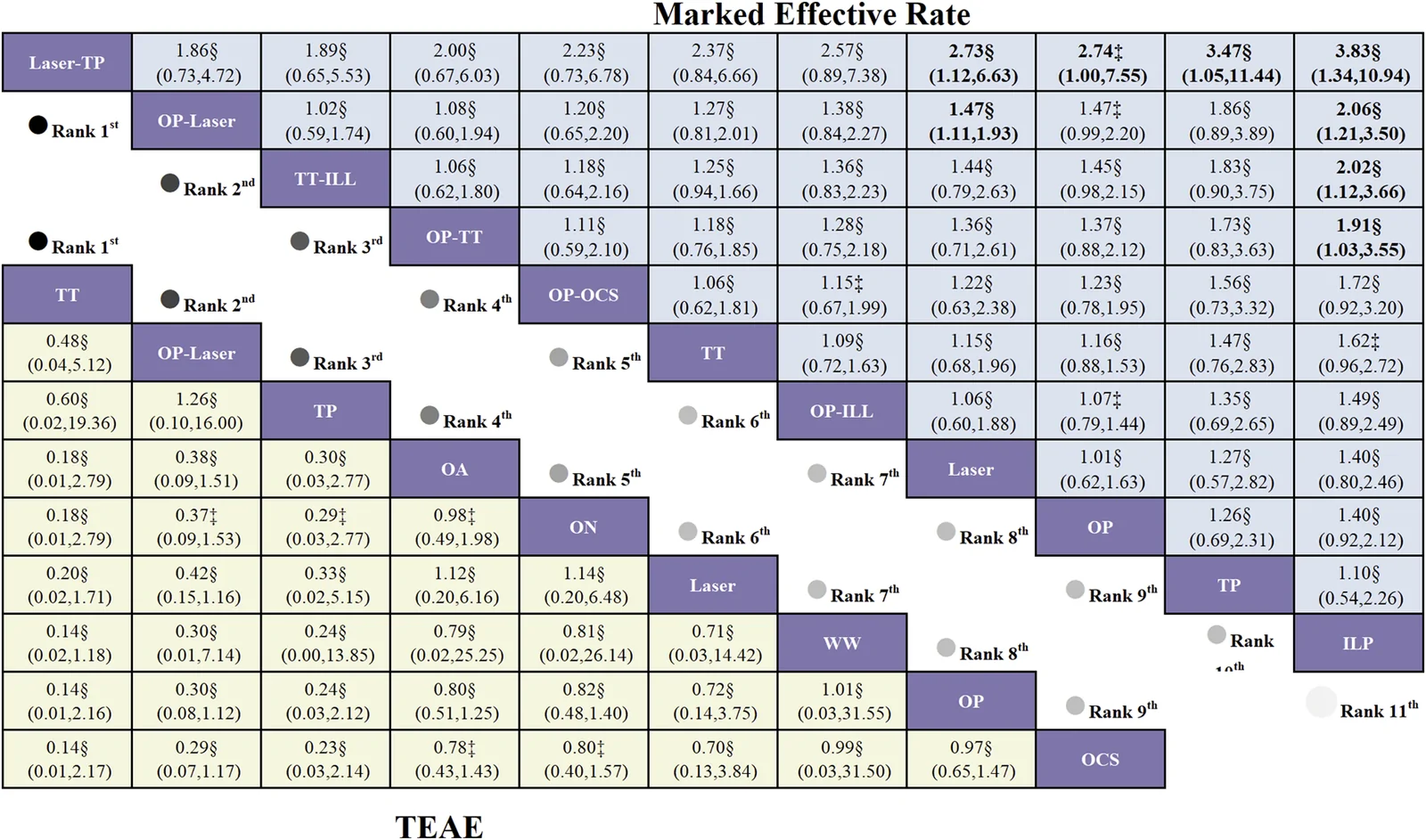
League matrix evaluating pharmacological, laser, and combined interventions for IH. Comparative estimates regarding Treatment-Emergent Adverse Events are detailed in the bottom-left section (highlighted in yellow), whereas the relative effects for the Marked Effective Rate are displayed in the top-right section (shaded in blue). Based on the GRADE framework, evidence certainty levels are marked as: * (high), † (moderate), ‡ (low), § (very low).

Twelve studies (887 patients) evaluated marked response rates across 11 intervention arms (Figure 3B). Compared to OP alone, Laser-TP was associated with a higher marked response rate, although the estimate was imprecise and supported by low-certainty evidence (RR = 2.74, 95% CI 1.00–7.55; low certainty) (Figure 5). Comprehensive ranking metrics identified Laser-TP had the highest SUCRA value (SUCRA = 94.6%; PrBest = 82.2%; mean rank = 1.5), followed by OP-Laser (SUCRA = 76.4%) (Supplementary Figure S4).

**FIGURE 6:**
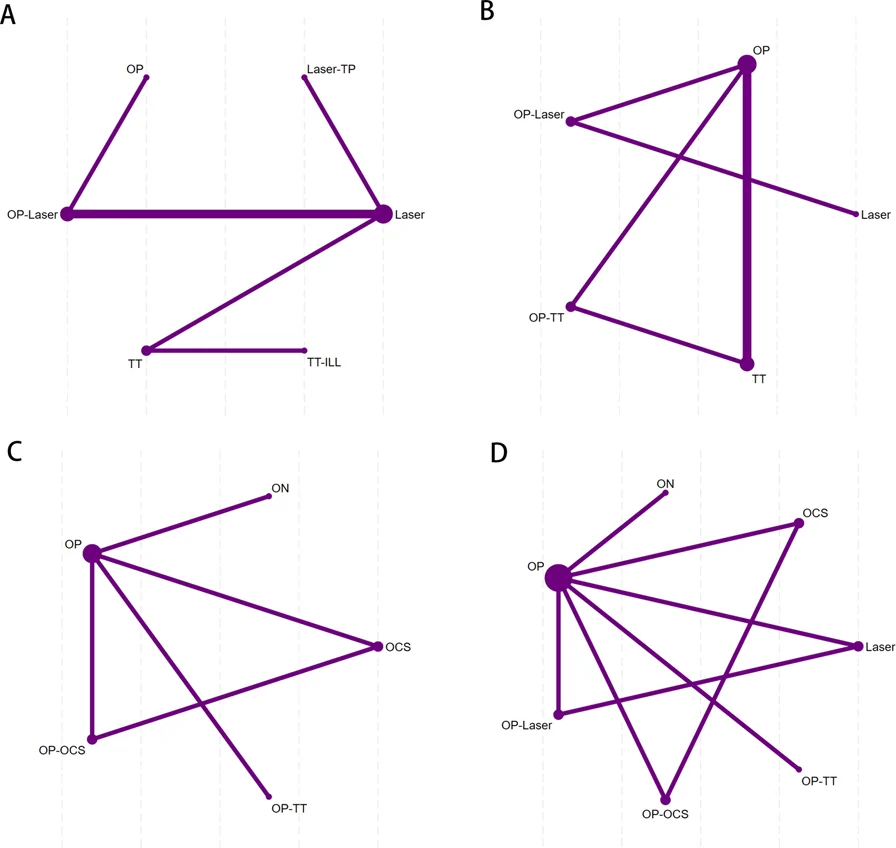
Network maps showing connections between pharmacologic, laser, and combination therapies for IH: **(A)** Total Effective Rate; **(B)** Marked Effective Rate for superficial IH; **(C)** VAS(Color); **(D)** VAS(Size).

Six studies (535 patients) reported total response rates across six interventions (Figure 6A). While OP-Laser yielded a higher overall response rate than OP alone (RR = 1.21, 95% CI 1.05–1.39; moderate certainty) (Figure 4A), global ranking metrics ultimately favored Laser-TP had the highest SUCRA value (SUCRA = 83.2%; PrBest = 67.2%; mean rank = 1.8). OP-Laser ranked second overall (SUCRA = 70.1%; mean rank = 2.5), followed by TT-ILL (Supplementary Figure S5).

###### 3.4.1.1.3 VAS scores

**FIGURE 7:**
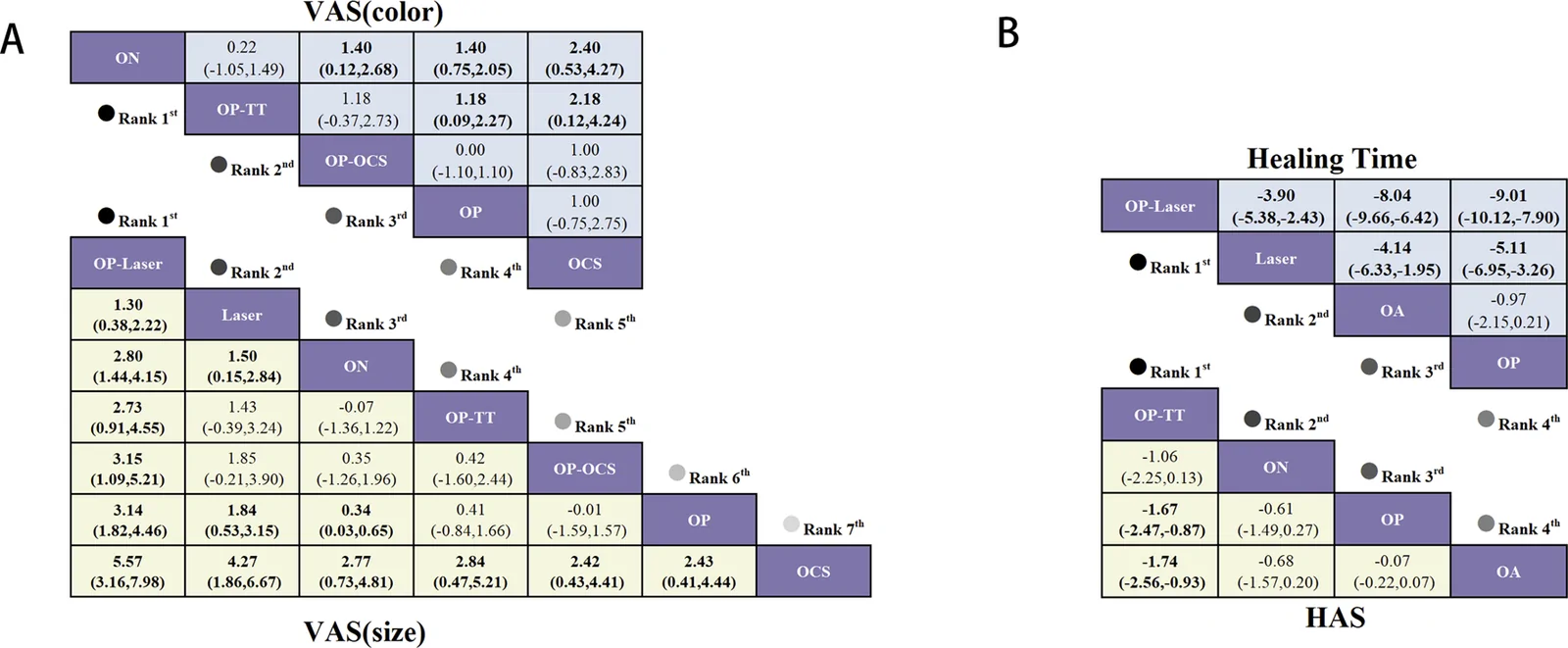
League matrix evaluating pharmacological, laser, and combined interventions for IH. **(A)** Comparative estimates regarding VAS (size) are detailed in the bottom-left section (highlighted in yellow), whereas the relative effects for VAS (color) are displayed in the top-right section (shaded in blue). **(B)** Similarly, the treatment effects concerning the HAS occupy the lower-left area (yellow shading), with the upper-right area (blue shading) illustrating the outcomes for Healing time. Based on the GRADE framework, evidence certainty levels are marked as: * (high), † (moderate), ‡ (low), and § (very low).

VAS scores were assessed across two dimensions: color and size. Three studies (132 patients, five interventions) reported VAS (color) outcomes (Figure 6C). Compared to OP alone, higher VAS color scores were observed with ON (MD = 1.40, 95% CI 0.12–2.68) and OP-TT (MD = 1.18, 95% CI 0.09–2.27) (Figure 7A). Comprehensive ranking metrics consistently identified ON had the highest SUCRA value (SUCRA = 90.4%; PrBest = 62.8%; mean rank = 1.4), sequentially followed by OP-TT (SUCRA = 81.4%; mean rank = 1.7) (Supplementary Figure S6). For VAS (size), four studies (229 patients) evaluated seven interventions (Figure 6D). OP-Laser (MD = 3.14, 95% CI 1.82–4.46), Laser (MD = 1.84, 95% CI 0.53–3.15), and ON (MD = 0.34, 95% CI 0.03–0.65) all associated with higher scores than OP (Figure 7B). OP-Laser ranked highest in the SUCRA analysis (SUCRA = 99.9%; PrBest = 99.2%; mean rank = 1.0), with Laser ranking second (SUCRA = 81.3%; mean rank = 2.1) (Supplementary Figure S7).

##### 3.4.1.2 Disease activity and progression

###### 3.4.1.2.1 HAS

**FIGURE 8:**
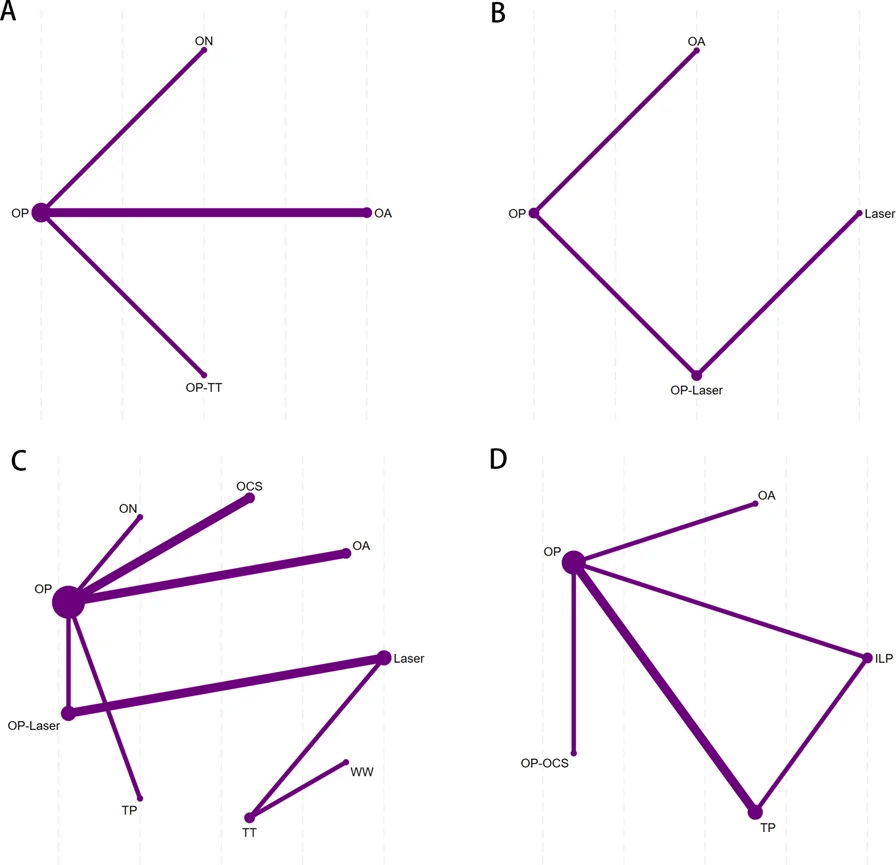
Network maps showing connections between pharmacologic, laser, and combination therapies for IH: **(A)** HAS; **(B)** Healing Time; **(C)** TEAE; **(D)** Recurrence Rate.

Four studies (513 patients, four interventions) evaluated changes in the HAS (Figure 8A). Compared to OP alone, OP-TT yielded a significant reduction in HAS (MD = −1.67, 95% CI -2.47 to −0.87) (Figure 7B). OP-TT ranked highest in the SUCRA analysis (SUCRA = 98.7%; PrBest = 96.1%; mean rank = 1.0), followed by ON (SUCRA = 63.0%; mean rank = 2.1) (Supplementary Figure S8).

###### 3.4.1.2.2 Healing time

Three studies (236 patients, four interventions) evaluated time to healing time (Figure 8B). Compared to OP alone, both OP-Laser (MD = −9.01 months, 95% CI -10.12 to −7.90) and Laser (MD = −5.11 months, 95% CI -6.95 to −3.26) were associated with shorter healing time (Figure 7B). OP-Laser ranked highest in the SUCRA analysis (SUCRA = 100%; PrBest = 100%; mean rank = 1.0), sequentially followed by Laser monotherapy (mean rank = 2.0). Given the limited number of included studies for this outcome, the results should be considered purely exploratory (Supplementary Figure S9).

#### 3.4.2 Safety assessments

##### 3.4.2.1 Treatment emergent adverse events

Eleven studies (996 patients, nine interventions) reported on treatment-emergent adverse events (Figure 8C). Compared to OP alone, TT (RR = 0.14, 95% CI 0.01–2.16) and OP-Laser (RR = 0.30, 95% CI 0.08–1.12) showed the lower point estimates for risk, though these reductions were not statistically significant and demonstrated wide confidence intervals (Figure 5). In the SUCRA analysis for adverse events, TT had the highest ranking for lower adverse-event risk (SUCRA = 85.5%; PrBest = 51.6%; mean rank = 2.2). While OP-Laser achieved the second-most favorable SUCRA (77.0%) and mean rank (2.8), rankogram analysis indicated that TT held a higher absolute probability of being ranked as the treatment with the lowest adverse-event risk (51.6%) (Supplementary Figure S10). However, because these rankings are derived from statistically non-significant differences and limited event data, they should be interpreted with caution.

##### 3.4.2.2 Recurrence rate

Four studies (510 patients, 5 interventions) reported on recurrence rates (Figure 8D). Compared to OP, OA yielded a lower point estimate for recurrence (RR = 0.64, 95% CI 0.32–1.28; moderate certainty), though this reduction was not statistically significant (Figure 4B). Global ranking metrics were closely split between OA and topical propranolol (TP). While OA achieved the highest SUCRA (74.5%) and most favorable mean rank (2.0), rankogram analysis indicated that TP held a higher absolute probability of being ranked first for minimizing recurrence (43.3% vs. 35.9% for OA) (Supplementary Figure S11). Similar to the safety outcomes, these recurrence rankings are based on non-significant differences from a limited number of studies, precluding definitive conclusions regarding superiority.

### 3.5 Subgroup analysis

In a subgroup analysis restricted to superficial IHs, OP-Laser significantly improved marked response compared to OP alone (RR = 1.81, 95% CI 1.27–2.57; moderate certainty) (Figures 4B and 6B). Comprehensive ranking metrics showed OP-Laser a favorable ranking in this superficial cohort (SUCRA = 96.3%; PrBest = 86.2%; mean rank = 1.1), distantly followed by OP-TT (SUCRA = 59.5%; mean rank = 2.6) (Supplementary Figure S12).

### 3.6 Meta-regression, sensitivity analysis, and publication bias

Baseline characteristics were well balanced across treatment arms. Network meta-regression demonstrated that while patient age did not modify basic cure or marked effective rates, it was a significant effect modifier for TEAE associated with OA therapy (Supplementary Tables S22–S24).

Leave-one-out sensitivity analyses confirmed the robustness of the primary findings; iterative removal of individual studies did not alter the direction, magnitude, or statistical significance of the pooled effects compared to OP (Supplementary Tables S18–S21).

Due to insufficient study counts for the remaining outcomes, comparison-adjusted funnel plots were generated only for marked effective rates and TEAE. Visual inspection revealed symmetrical distributions, indicating a low risk of publication bias or small-study effects (Supplementary Figures S1 and S2).

## 4 Discussion

To address the scarcity of head-to-head trials across a crowded therapeutic landscape, we performed a network meta-analysis evaluating the relative efficacy and safety of 16 interventions for IH. Synthesizing data from 25 randomized controlled trials (n = 2,044), this analysis provides moderate-certainty evidence that, compared to OP, TT-ILL ranked favorably for achieving >75% tumor involution. Furthermore, moderate-to high-certainty evidence suggests that OP combined with either oral corticosteroids or intralesional bleomycin may represent efficacious alternatives, with higher excellent response rates than OP monotherapy. Adding laser therapy to propranolol was associated with improved both marked and total response rates compared with propranolol monotherapy (low-to moderate-certainty evidence). While OA demonstrated a trend toward reduced recurrence relative to OP, this difference did not reach statistical significance (moderate certainty). Notably, within the superficial IH subgroup, combined laser regimens were associated with higher marked response rates than OP in achieving marked responses (moderate certainty). The evidence base for all remaining interventions was of low or very low certainty.

Given its established role as the first-line standard of care for IH, oral propranolol served as the reference intervention in this NMA. While early studies attributed its pleiotropic effects to vasoconstriction, apoptosis induction, nitric oxide reduction, and modulation of the renin-angiotensin system (Munabi et al., 2016; Lee et al., 2021; Stiles et al., 2012; Wong et al., 2012; Wnęk et al., 2017; Sharifpanah et al., 2014; Holm et al., 2024), recent data highlight a distinct mechanism. The R(+) enantiomer, which lacks β-blocking activity, directly inhibits the endothelial transcription factor SOX18. This inhibition downregulates the mevalonate pathway and suppresses cholesterol biosynthesis, ultimately depriving endothelial cells of the lipid substrates required for proliferation. Furthermore, Li et al. demonstrated that the drug modulates angiogenic protein networks, restoring vascular homeostasis by rebalancing pro- and anti-angiogenic factors (Iruela-Arispe, 2025; Liang et al., 2025). Correspondingly, Chen et al. observed that IH progression relies on the dynamic turnover of dominant cell subpopulations, enabling propranolol to act across multiple cellular lineages (Chen et al., 2026). As anticipated, oral propranolol provided a reliable clinical benchmark in our analysis, showing consistent efficacy across all evaluated endpoints.

Treatment rankings should be interpreted with caution. SUCRA values provide a probabilistic summary of relative ranking, but they do not directly reflect the magnitude, precision, or certainty of treatment effects. In this network, some highly ranked interventions were supported by sparse data, limited direct comparisons, or imprecise estimates. Therefore, we interpreted SUCRA rankings alongside the corresponding effect estimates, 95% confidence intervals, and CINeMA certainty ratings, rather than as standalone evidence of treatment superiority.

Among the evaluated interventions, topical and multi-target combination regimens showed favorable efficacy signals for selected outcomes. Notably, TT-ILL ranked among the most efficacious regimens, driving a >75% reduction in tumor volume. This profound response likely stems from the synergistic dual action of localized β-adrenergic blockade and potent sclerotherapy. For superficial IHs, topical timolol offers comparable efficacy to oral propranolol but with a superior safety profile (Huang et al., 2024). Histologically, lauromacrogol directly induces endothelial injury, intimal denudation, and subendothelial collagen exposure, precipitating localized thrombosis and venous fibrosis (Yang et al., 2018b). This aggressive physio-pharmacological occlusion accounts for the regimen’s exceptional capacity to drive rapid volumetric reduction.

Alternatively, systemic corticosteroids and intralesional bleomycin amplify therapeutic outcomes *via* distinct pathways: molecular regulation and direct cytotoxicity. Corticosteroids potently suppress VEGF-A expression to disrupt neovascularization (Greenberger et al., 2010), whereas bleomycin induces targeted endothelial damage and focal fibrosis, directly precipitating lesion regression (Muir et al., 2004). These underlying mechanisms elucidate the striking efficacy profiles of OP-OCS and OP-ILB; robust evidence confirms that both combinations yield significantly higher excellent response rates compared to oral propranolol monotherapy.

The favorable VAS outcomes of OP-TT and ON are consistent with established pathophysiological and pharmacokinetic models of IH. While systemic propranolol halts deep tissue proliferation and drives gross volumetric shrinkage, the addition of topical timolol ensures high-concentration drug penetration at the epidermal layer. This accelerated clearance of superficial erythema minimizes the risk of late-stage aesthetic sequelae (Huang et al., 2024; Ge et al., 2016). Furthermore, nadolol, a hydrophilic non-selective β-blocker, possesses a significantly longer half-life than propranolol43, ensuring continuous, steady-state receptor blockade. This uninterrupted inhibition drives a more uniform and exhaustive involution, significantly reducing the incidence of irreversible fibrofatty residuum and redundant skin (Munabi et al., 2016; Wong et al., 2012). However, the available evidence for these outcomes was based on a limited number of studies. Therefore, the apparent aesthetic advantages of OP-TT and ON should be interpreted cautiously.

Laser modalities may improve selected clinical outcomes *via* precise photothermal effects. PDL arrests lesion growth by inducing intravascular microthrombosis, whereas long-pulsed Nd:YAG lasers target hemoglobin and oxyhemoglobin, converting light to thermal energy that directly ablates the vascular wall (Ziad et al., 2023). The synergy between this targeted thermal coagulation and the anti-angiogenic properties of propranolol explains why laser-combined regimens more readily achieve >50% volumetric reduction in superficial IH.

Atenolol—a hydrophilic β-blocker unable to cross the blood-brain barrier—delivers equivalent vascular inhibition while sparing patients potential neurocognitive burdens. Collectively, these synergistic effects stabilize the lesion microenvironment, optimizing long-term pediatric outcomes and demonstrating a trend toward a reduced risk of rebound growth (Shi et al., 2025).

The absence of surgery from the quantitative network should not be interpreted as evidence that surgical treatment lacks clinical value. Rather, it reflects the absence of eligible randomized controlled trials comparing surgical excision with the interventions included in this analysis. In the current beta-blocker era, surgery is no longer a routine first-line option for most proliferating IHs, but it remains a selective complementary option for residual fibrofatty tissue or contour deformity after involution, treatment-refractory lesions, contraindications to medical therapy, selected ulcerated or bleeding lesions amenable to resection, and cases requiring functional or cosmetic reconstruction (Beqo et al., 2023).

Overall, our findings suggest that selected combination regimens and alternative pharmacotherapies may offer clinical advantages over OP monotherapy for specific outcomes and patient subgroups. By synthesizing a historically fragmented evidence base, this network meta-analysis provides actionable comparative data to support phenotype-driven treatment selection. Therapeutic selection in IH must be rigorously stratified by clinical objectives. For OP-intolerant patients, TT-ILL may be considered as a potentially effective option for selected lesions when excellent response is the primary objective. OP-OCS and OP-ILB may provide higher excellent response rates than OP alone, although their use should be balanced against safety considerations and clinical indication. OP-TT or ON may be relevant when aesthetic improvement is a major treatment goal, whereas OA may be considered for patients in whom propranolol tolerability is a concern. For superficial IHs, laser-combined regimens may provide additional benefit, but these findings require cautious interpretation because of heterogeneity in laser protocols and limited direct evidence.

Our methodology expands upon previous network meta-analyses (Yang et al., 2020; Hu et al., 2026) by treating individual pharmacotherapies as discrete nodes and explicitly evaluating combination regimens. Prior syntheses have generally either clustered non-propranolol agents into a single comparative node (Hu et al.) or excluded combination therapies entirely (Yang et al.). This structural refinement permitted a more precise isolation of treatment effects on specific visual endpoints, such as lesion color and size. Regarding safety and long-term control, Chen et al. (Chen et al., 2023) previously reported a higher incidence of adverse events with propranolol compared to atenolol. Although our pooled analysis did not detect a statistically significant difference in overall adverse-event rates between the two, this should not be interpreted as evidence of equivalent safety because the available safety data were limited and may have been underpowered to detect uncommon adverse events. Similarly, although atenolol showed a favorable point estimate for recurrence, the difference was not statistically significant. For the management of superficial IH, Lin et al. (Lin et al., 2020) proposed that topical beta-blockers might replace OP as the standard of care. However, our subgroup data suggested that OP combined with laser therapy may achieve favorable marked response rates in this population. This finding should also be interpreted cautiously given the limited number of studies and heterogeneity of laser-based protocols. In addition, our study expands upon prior evidence syntheses by integrating validated continuous clinical metrics, namely, HAS and VAS, while analyzing them as separate outcomes because of differences in scale direction and clinical interpretation.

A primary strength of this analysis is the integration of minimal clinically important difference (MCID) thresholds and the CINeMA framework, which grounds our effect estimates in actionable clinical relevance rather than mere statistical significance. Furthermore, by evaluating individual pharmacotherapies as discrete nodes rather than clustering conceptually heterogeneous treatments, we preserved the granularity necessary to isolate specific therapeutic effects. However, these findings must be interpreted within the context of several limitations. The inherent ethical and practical challenges of blinding in infant trials resulted in many studies carrying an elevated risk of bias, warranting caution when interpreting comparisons supported by sparse data. Additionally, while our visual assessments for publication bias were unremarkable, the limited number of trials per node restricts our statistical power to definitively exclude small-study effects. Furthermore, the overall sample sizes and low event rates across the included trials limited our statistical power to detect meaningful differences in secondary outcomes, particularly uncommon adverse events and recurrence rates. Consequently, the absence of statistically significant differences in these safety and recurrence profiles should not be strictly interpreted as evidence of clinical equivalence. Most importantly, variable outcome definitions across the primary literature—particularly concerning treatment response definitions, adverse events and subjective clinical scoring—introduced methodological heterogeneity. Although we used strict prespecified thresholds to harmonize response outcomes, with excellent response, marked response, and total response defined as >75%, >50%, and >25% reduction in tumor size, respectively, residual heterogeneity may remain because the included trials differed in their assessment methods, including clinical estimation of lesion area, photographic assessment, ultrasound- or MRI-based volume measurement, and the incorporation of color fading into response grading. This may have influenced the precision of the network estimates and treatment rankings, particularly for response-based outcomes. This was statistically reflected by borderline incoherence (P < 0.10) within certain closed loops, which we proactively accounted for by downgrading the certainty of evidence for those specific networks. Another limitation is the possibility of transitivity violations across treatment nodes. Although the included trials were broadly comparable in enrolling infants with clinically relevant infantile hemangiomas during the early treatment window, several key effect modifiers were not completely balanced across comparisons. In particular, trials of topical β-blockers and watchful waiting tended to include smaller or superficial lesions, whereas systemic β-blockers, corticosteroids, intralesional agents, and combination regimens were more commonly evaluated in problematic, mixed, deep, or high-risk lesions. Differences in baseline lesion burden, anatomical location, age at treatment initiation, treatment duration, and follow-up may have influenced the relative treatment effects. Because some outcome networks lacked closed loops, formal inconsistency assessment was not feasible for all outcomes. Therefore, indirect comparisons and treatment rankings, especially those involving topical or local therapies *versus* systemic or combination regimens, should be interpreted cautiously. In addition to these potential between-node differences, within-node heterogeneity should also be considered when interpreting comparative efficacy. Although treatment nodes were defined according to the active agent, route of administration, and treatment strategy, some nodes, particularly laser-based therapies and combination regimens, may still include clinically relevant variation. Laser-based nodes varied in laser modality, wavelength, fluence, pulse duration, treatment interval, and number of sessions. Combination regimens also differed in the dose, timing, sequence, and duration of each component. Therefore, the estimated effects for these nodes should be interpreted as average effects of broadly similar treatment strategies rather than as evidence for a single standardized protocol. This is particularly relevant for treatment rankings, which should be interpreted together with effect estimates, confidence intervals, and certainty of evidence.

## 5 Conclusion

Our comprehensive comparative analysis demonstrates that several multimodal or alternative regimens may provide additional efficacy benefits over traditional oral propranolol monotherapy for selected outcomes in the management of IH. Specifically, some combination regimens involving localized sclerosing agents or systemic adjunctive therapy, such as TT-ILL, OP-OCS, and OP-ILB, showed favorable effect estimates or treatment rankings for certain efficacy endpoints. That said, the ethical and logistical barriers inherent to pediatric trials have resulted in a sparse evidence base, currently precluding the identification of a definitively optimal combination strategy. Readers must interpret these comparative benefits with measured caution, as the underlying literature remains constrained by notable methodological vulnerabilities—chiefly, low-certainty evidence for specific modalities, unblinded designs, underpowered cohorts, and highly heterogeneous treatment parameters. Ultimately, definitively mapping the risk-benefit profiles of these interventions will require large-scale, multicenter randomized controlled trials governed by standardized therapeutic protocols.

## Data Availability

The original contributions presented in the study are included in the article/Supplementary Material, further inquiries can be directed to the corresponding author.
