# Supplementary material for: Comparative efficacy and safety of pharmacologic, laser, and combination therapies for infantile hemangioma: a systematic review and network meta-analysis with evidence certainty assessment
Source: Front Pharmacol. 2026 Sep 9;17:1882566. doi: 10.3389/fphar.2026.1882566 (PMC13597854; doi:10.3389/fphar.2026.1882566)
Supplement: Supplementary file 1 [file Table1.docx]

Supplementary Material

| **Table of Contents** | | |
| --- | --- | --- |
| Title | Content | page |
| Table S1 | PRISMA NMA Checklist of Items to Include When Reporting a Systematic Review Involving a Network Meta-analysis | 2-5 |
| Table S2 | Literature Search Strategy | 5-9 |
| Table S3 | Global inconsistency table for Marked Effective Rate and Recurrence Rate | 9 |
| Table S4 | Node-splitting analysis for the Marked Effective Rate outcome in IH | 9-10 |
| Table S5 | Node-splitting analysis for the Recurrence Rate outcome in IH | 10 |
| Table S6 | Loop-specific inconsistency test table for the Marked Effective Rate outcome in IH | 10 |
| Table S7 | Loop-specific inconsistency test table for the Recurrence Rate outcome in IH | 10 |
| Table S8 | SUCRA ranking table for the Excellent Response Rate outcome in IH | 10 |
| Table S9 | SUCRA ranking table for the Marked Effective Rate outcome in IH | 11 |
| Table S10 | SUCRA ranking table for the Total Effective Rate outcome in IH | 11 |
| Table S11 | SUCRA ranking table for the VAS (Color) outcome in IH | 11 |
| Table S12 | SUCRA ranking table for the VAS (Size) outcome in IH | 12 |
| Table S13 | SUCRA ranking table for the HAS outcome in IH | 12 |
| Table S14 | SUCRA ranking table for the Healing Time outcome in IH | 12 |
| Table S15 | SUCRA ranking table for the TEAE outcome in IH | 12-13 |
| Table S16 | SUCRA ranking table for the Recurrence Rate outcome in IH | 13 |
| Table S17 | SUCRA ranking table for the Marked Effective Rate outcome in Superficial IH | 13 |
| Table S18 | Sensitivity analysis for the Excellent Response Rate outcome in IH | 13-14 |
| Table S19 | Sensitivity analysis for the Marked Effective Rate outcome in IH | 14-16 |
| Table S20 | Sensitivity analysis for the Total Effective Rate outcome in IH | 16-17 |
| Table S21 | Sensitivity analysis for the TEAE outcome in IH | 17-18 |
| Table S22 | Meta-regression analysis of the Excellent Response Rate outcome with mean age as a moderator in IH | 19 |
| Table S23 | Meta-regression analysis of the Marked Effective Rate outcome with mean age as a moderator in IH | 19 |
| Table S24 | Meta-regression analysis of the TEAE outcome with mean age as a moderator in IH | 19-20 |
| Figure S1 | Comparison-adjusted funnel plot for the Marked Effective Rate outcome in IH | 20 |
| Figure S2 | Comparison-adjusted funnel plot for the TEAE outcome in IH | 21 |
| Table S25 | GRADE certainty of evidence for the Excellent Response Rate outcome in IH | 21-22 |
| Table S26 | GRADE certainty of evidence for the Marked Effective Rate outcome in IH | 22-23 |
| Table S27 | GRADE certainty of evidence for the Total Effective Rate outcome in IH | 23-24 |
| Table S28 | GRADE certainty of evidence for the TEAE outcome in IH | 24-25 |
| Table S29 | GRADE certainty of evidence for the Recurrence Rate outcome in IH | 25 |
| Table S30 | GRADE certainty of evidence for the Marked Effective Rate outcome in superficial IH | 25-26 |
| Figure S3 | SUCRA ranking plot table for the Excellent Response Rate outcome in IH | 26 |
| Figure S4 | SUCRA ranking plot table for the Marked Effective Rate outcome in IH | 27 |
| Figure S5 | SUCRA ranking plot table for the Total Effective Rate outcome in IH | 27 |
| Figure S6 | SUCRA ranking plot table for the VAS (Color) outcome in IH | 28 |
| Figure S7 | SUCRA ranking plot table for the VAS (Size) outcome in IH | 28 |
| Figure S8 | SUCRA ranking plot table for the HAS outcome in IH | 29 |
| Figure S9 | SUCRA ranking plot table for the Healing time outcome in IH | 29 |
| Figure S10 | SUCRA ranking plot table for the TEAE outcome in IH | 30 |
| Figure S11 | SUCRA ranking plot table for the Recurrence Rate outcome in IH | 30 |
| Figure S12 | SUCRA ranking plot for the Marked Effective Rate outcome in Superficial IH | 31 |

Supplementary Table 1	PRISMA NMA Checklist of Items to Include When Reporting a Systematic Review Involving a Network Meta-analysis

| Section/Topic | Item # | Checklist Item | Reported on Page # |
| --- | --- | --- | --- |
| TITLE |  |  |  |
| Title | 1 | Identify the report as a systematic review *incorporating*  A network meta-analysis (or related form of meta-analysis). | 1 |
|  |  |  |  |
| ABSTRACT |  |  |  |
| Structured summary | 2 | Provide a structured summary including, as applicable:  **Background:** main objectives  **Methods:** data sources; study eligibility criteria, participants, and interventions; study appraisal; and *synthesis methods, such as network meta-analysis.*  **Results:** number of studies and participants identified; summary estimates with corresponding confidence/credible intervals; treatment rankings may also be discussed. Authors may choose to summarize pairwise comparisons against a chosen treatment included in their analyses for brevity.  **Discussion/Conclusions:** limitations; conclusions and implications of findings.  **Other:** systematic review registration number with registry name. | 1-2 |
|  |  |  |  |
| INTRODUCTION |  |  |  |
| Rationale | 3 | Describe the rationale for the review in the context of what is already known*, including mention of why a network meta-analysis has been conducted.* | 2 |
| Objectives | 4 | Provide an explicit statement of questions being addressed, with reference to participants, interventions, comparisons, outcomes, and study design (PICOS). | 3 |
|  |  |  |  |
| METHODS |  |  |  |
| Protocol and registration | 5 | Indicate whether a review protocol exists and if and where it can be accessed (e.g., Web address); and, if available, provide registration information, including registration number. | 3 |
| Eligibility criteria | 6 | Specify study characteristics (e.g., PICOS, length of follow-up) and report characteristics (e.g., years considered, language, publication status) used as criteria for eligibility, giving rationale. *Clearly describe eligible treatments included in the treatment network, and note whether any have been clustered or merged into the same node (with justification).* | 3 |
| Information sources | 7 | Describe all information sources (e.g., databases with dates of coverage, contact with study authors to identify additional studies) in the search and date last searched. | 3 |
| Search | 8 | Present full electronic search strategy for at least one database, including any limits used, such that it could be repeated. | 3，Supplementary  TableS2 |
| Study selection | 9 | State the process for selecting studies (i.e., screening, eligibility, included in systematic review, and, if applicable, included in the meta-analysis). | 3 |
| Data collection process | 10 | Describe method of data extraction from reports (e.g., piloted forms, independently, in duplicate) and any processes for obtaining and confirming data from investigators. | 3 |
| Data items | 11 | List and define all variables for which data were sought (e.g., PICOS, funding sources) and any assumptions and simplifications made. | 3 |
| Geometry of the network | S1 | Describe methods used to explore the geometry of the treatment network under study and potential biases related to it. This should include how the evidence base has been graphically summarized for presentation, and what characteristics were compiled and used to describe the evidence base to readers. | 4 |
| Risk of bias within individual studies | 12 | Describe methods used for assessing risk of bias of individual studies (including specification of whether this was done at the study or outcome level), and how this information is to be used in any data synthesis. | 4 |
| Summary measures | 13 | State the principal summary measures (e.g., risk ratio, difference in means). Also describe the use of additional summary measures assessed, such as treatment rankings and surface under the cumulative ranking curve (SUCRA) values, as well as modified approaches used to present summary findings from meta-analyses. | 4 |
| Planned methods of analysis | 14 | Describe the methods of handling data and combining results of studies for each network meta-analysis. This should include, but not be limited to:   - Handling of multi-arm trials; - Selection of variance structure; - Selection of prior distributions in Bayesian analyses; and - Assessment of model fit. | 4 |
| Assessment of Inconsistency | S2 | Describe the statistical methods used to evaluate the agreement of direct and indirect evidence in the treatment network(s) studied. Describe efforts taken to address its presence when found. | 4 |
| Risk of bias across studies | 15 | Specify any assessment of risk of bias that may affect the cumulative evidence (e.g., publication bias, selective reporting within studies). | 4 |
| Additional analyses | 16 | Describe methods of additional analyses if done, indicating which were pre-specified. This may include, but not be limited to, the following:   - Sensitivity or subgroup analyses; - Meta-regression analyses; - Alternative formulations of the treatment network; and - Use of alternative prior distributions for Bayesian analyses (if applicable). | 4 |
| RESULTS† |  |  |  |
| Study selection | 17 | Give numbers of studies screened, assessed for eligibility, and included in the review, with reasons for exclusions at each stage, ideally with a flow diagram. | 5, Figure 1 |
| Presentation of network structure | S3 | Provide a network graph of the included studies to enable visualization of the geometry of the treatment network. | Figure 3-5 |
| Summary of network geometry | S4 | Provide a brief overview of characteristics of the treatment network. This may include commentary on the abundance of trials and randomized patients for the different interventions and pairwise comparisons in the network, gaps of evidence in the treatment network, and potential biases reflected by the network structure. | 5-8 |
| Study characteristics | 18 | For each study, present characteristics for which data were extracted (e.g., study size, PICOS, follow-up period) and provide the citations. | 5, Table 1 |
| Risk of bias within studies | 19 | Present data on risk of bias of each study and, if available, any outcome level assessment. | 5, Figure 2 |
| Results of individual studies | 20 | For all outcomes considered (benefits or harms), present, for each study: 1) simple summary data for each intervention group, and 2) effect estimates and confidence intervals. *Modified approaches may be needed to deal with information from larger networks.* | 5-8 |
| Synthesis of results | 21 | Present results of each meta-analysis done, including confidence/credible intervals. In larger networks, authors may focus on comparisons versus a particular comparator (e.g. placebo or standard care), with full findings presented in an appendix. League tables and forest plots may be considered to summarize pairwise comparisons. If additional summary measures were explored (such as treatment rankings), these should also be presented. | 5-8 |
| Exploration for inconsistency | S5 | Describe results from investigations of inconsistency. This may include such information as measures of model fit to compare consistency and inconsistency models, *P* values from statistical tests, or summary of inconsistency estimates from different parts of the treatment network. | 7 |
| Risk of bias across studies | 22 | Present results of any assessment of risk of bias across studies for the evidence base being studied. | 7, Supplementary Table 27-32 |
| Results of additional analyses | 23 | Give results of additional analyses, if done (e.g., sensitivity or subgroup analyses, meta-regression analyses*, alternative network geometries studied, alternative choice of prior distributions for Bayesian analyses,* and so forth). | 7-8 |
|  |  |  |  |
| DISCUSSION |  |  |  |
| Summary of evidence | 24 | Summarize the main findings, including the strength of evidence for each main outcome; consider their relevance to key groups (e.g., healthcare providers, users, and policy-makers). | 8-9 |
| Limitations | 25 | Discuss limitations at study and outcome level (e.g., risk of bias), and at review level (e.g., incomplete retrieval of identified research, reporting bias). *Comment on the validity of the assumptions, such as transitivity and consistency. Comment on any concerns regarding network geometry (e.g., avoidance of certain comparisons).* | 10 |
| Conclusions | 26 | Provide a general interpretation of the results in the context of other evidence, and implications for future research. | 10 |
|  |  |  |  |
| FUNDING |  |  |  |
| Funding | 27 | Describe sources of funding for the systematic review and other support (e.g., supply of data); role of funders for the systematic review. This should also include information regarding whether funding has been received from manufacturers of treatments in the network and/or whether some of the authors are content experts with professional conflicts of interest that could affect use of treatments in the network. | 10 |

PICOS = population, intervention, comparators, outcomes, study design.

* Text in italics indicate S wording specific to reporting of network meta-analyses that has been added to guidance from the PRISMA statement.

† Authors may wish to plan for use of appendices to present all relevant information in full detail for items in this section.

| Supplementary Table 2. Literature Search Strategy | |
| --- | --- |
| Pubmed | Num Search Hits  #1 "Hemangioma, Capillary"[Mesh] 3371  #2 ((((Hemangioma, Capillary[Title/Abstract])) OR (Capillary Hemangioma[Title/Abstract])) OR (Capillary Hemangiomas[Title/Abstract])) OR (Hemangiomas, Capillary[Title/Abstract]) 1670  #3 "Infant"[Mesh] 1337532  #4 ((infant[Title/Abstract]) OR (infants[Title/Abstract])) OR ("Infant"[Mesh]) 1467950  #5 ((((infantile hemangioma[Title/Abstract]) ) OR (infantile haemangioma[Title/Abstract])) OR (capillary infantile hemangioma[Title/Abstract])) 1812  #6 ("Hemangioma, Capillary"[Mesh]) OR (((((Hemangioma, Capillary[Title/Abstract])) OR (Capillary Hemangioma[Title/Abstract])) OR (Capillary Hemangiomas[Title/Abstract])) OR (Hemangiomas, Capillary[Title/Abstract])) 4429  #7 (((infant[Title/Abstract]) OR (infants[Title/Abstract])) OR ("Infant"[Mesh])) AND (("Hemangioma, Capillary"[Mesh]) OR (((((Hemangioma, Capillary[Title/Abstract])) OR (Capillary Hemangioma[Title/Abstract])) OR (Capillary Hemangiomas[Title/Abstract])) OR (Hemangiomas, Capillary[Title/Abstract]))) 952  #8 (((((infantile hemangioma[Title/Abstract]) ) OR (infantile haemangioma[Title/Abstract])) OR (capillary infantile hemangioma[Title/Abstract]))) OR ((((infant[Title/Abstract]) OR (infants[Title/Abstract])) OR ("Infant"[Mesh])) AND (("Hemangioma, Capillary"[Mesh]) OR (((((Hemangioma, Capillary[Title/Abstract])) OR (Capillary Hemangioma[Title/Abstract])) OR (Capillary Hemangiomas[Title/Abstract])) OR (Hemangiomas, Capillary[Title/Abstract])))) 2507  #9 "Adrenergic beta-Antagonists"[Mesh] 44429  #10 (((((((((((Adrenergic beta-Antagonist[Title/Abstract]) OR (Adrenergic beta Antagonist[Title/Abstract])) OR (Adrenergic beta-Receptor Blockader[Title/Abstract])) OR (Adrenergic beta Receptor Blockader[Title/Abstract])) OR (beta-Adrenergic Antagonist[Title/Abstract])) OR (beta Adrenergic Antagonist[Title/Abstract])) OR (beta-Adrenergic Antagonists[Title/Abstract])) OR (Propranolol[Title/Abstract])) OR (Atenolol[Title/Abstract])) OR (Nadolol[Title/Abstract])) OR (Timolol[Title/Abstract])) OR (Carteolol[Title/Abstract]) 47565  #11 ("Adrenergic beta-Antagonists"[Mesh]) OR ((((((((((((Adrenergic beta-Antagonist[Title/Abstract]) OR (Adrenergic beta Antagonist[Title/Abstract])) OR (Adrenergic beta-Receptor Blockader[Title/Abstract])) OR (Adrenergic beta Receptor Blockader[Title/Abstract])) OR (beta-Adrenergic Antagonist[Title/Abstract])) OR (beta Adrenergic Antagonist[Title/Abstract])) OR (beta-Adrenergic Antagonists[Title/Abstract])) OR (Propranolol[Title/Abstract])) OR (Atenolol[Title/Abstract])) OR (Nadolol[Title/Abstract])) OR (Timolol[Title/Abstract])) OR (Carteolol[Title/Abstract])) 81158  #12 "Glucocorticoids"[Mesh] 75453  #13 (((((Corticosteroids[Title/Abstract]) OR (Prednisone[Title/Abstract])) OR (Prednisolone[Title/Abstract])) OR (Triamcinolone[Title/Abstract])) OR (Betamethasone[Title/Abstract])) OR (steroid[Title/Abstract]) 319047  #14 ("Glucocorticoids"[Mesh]) OR ((((((Corticosteroids[Title/Abstract]) OR (Prednisone[Title/Abstract])) OR (Prednisolone[Title/Abstract])) OR (Triamcinolone[Title/Abstract])) OR (Betamethasone[Title/Abstract])) OR (steroid[Title/Abstract])) 367115  #15 Captopril[Title/Abstract] 12185  #16 ((Vincristine[Title/Abstract]) OR (Sirolimus[Title/Abstract])) OR (Interferon[Title/Abstract]) 220255  #17 "Lasers"[Mesh] 64378  #18 "Laser Therapy"[Mesh] 71756  #19 (((Laser[Title/Abstract]) OR (Pulsed Lasers[Title/Abstract])) OR (Pulsed Laser[Title/Abstract])) OR (Laser Therapies[Title/Abstract]) 356005  #20 (((Bleomycin[Title/Abstract]) OR (Polidocanol[Title/Abstract])) OR (surgery[Title/Abstract])) OR (excision[Title/Abstract]) 1,861,381  #21 "Sclerotherapy"[Mesh] 6,359  #22 #11 or #14 or #15 or #16 or #17 or #18 or #19 or #20 or #21 2838336  #23 #8 AND #22 1379  #24 #8 AND #22 Filters: Randomized Controlled Trial 26 |
|  |  |
|  |  |
|  |  |
|  |  |
|  |  |
|  |  |
| Web of Science | 1: TS=(Hemangioma, Capillary) 1875  2: TS=("Capillary Hemangioma" OR "Capillary Hemangiomas" OR "Hemangiomas, Capillary") 1467  3: TS=("infant" or "infants") 1337532  4: #1 or #2 1996  5: #3 and #4 167  6: TS=("infantile hemangioma" OR "infantile haemangioma" OR "capillary infantile hemangioma") 2013  7: #5 or #6 2139  8: TS=("Adrenergic beta-Antagonist" OR "Adrenergic beta Antagonist" OR "Adrenergic beta-Receptor Blockader" OR "Adrenergic beta Receptor Blockader" OR "beta-Adrenergic Antagonist" OR "beta Adrenergic Antagonist" OR "beta-Adrenergic Antagonists" OR "Propranolol" OR "Atenolol" OR "Nadolol" OR "Timolol" OR "Carteolol") 25725  9: TS=("Glucocorticoids" OR "Corticosteroids" OR "Prednisone" OR "Prednisolone" OR "Triamcinolone" OR "Betamethasone" OR "steroid") 286005  10:TS=("Captopril" OR "Vincristine" OR "Sirolimus" OR "Interferon") 251430  11: TS=("Lasers" OR "Laser Therapy" OR "Sclerotherapy" OR "Laser" OR "Pulsed Lasers" OR "Pulsed Laser" OR "Laser Therapies") 1055647  12: TS=("Bleomycin" OR "Polidocanol" OR "surgery" OR "excision") 1607644  13: TS=(random* OR randomi* OR randomly  OR placebo* OR sham  OR trial OR "clinical trial" OR "controlled clinical trial"  OR "double blind" OR "single blind" OR "triple blind"  OR crossover OR "cross over" OR "parallel group"  OR cluster random*  OR RCT) 4065798  14: #8 OR #9 OR #10 OR #11 OR #12 3144269  15: #7 AND #14 1300  16: #13 AND #15 148 |
|  |  |
|  |  |
|  |  |
|  |  |
|  |  |
|  |  |
|  |  |
| Cochrane | ID Search Hits  #1 MeSH descriptor: [Hemangioma, Capillary] explode all trees 54  #2 "Capillary Hemangioma" OR "Capillary Hemangiomas" OR "Hemangiomas, Capillary" 86  #3 MeSH descriptor: [Infant] explode all trees 46660  #4 infants 43395  #5 #1 or #2 121  #6 #3 or #4 70948  #7 #5 and #6 52  #8 "infantile hemangioma" OR "infantile haemangioma" OR "capillary infantile hemangioma" 114  #9 MeSH descriptor: [Adrenergic beta-Antagonists] explode all trees 5634  #10 "Adrenergic beta-Antagonist" OR "Adrenergic beta Antagonist" OR "Adrenergic beta-Receptor Blockader" OR "Adrenergic beta Receptor Blockader" OR "beta-Adrenergic Antagonist" OR "beta Adrenergic Antagonist" OR "beta-Adrenergic Antagonists" OR "Propranolol" OR "Atenolol" OR "Nadolol" OR "Timolol" OR "Carteolol" 11225  #11 MeSH descriptor: [Glucocorticoids] explode all trees 6033  #12 "Corticosteroids" OR "Prednisone" OR "Prednisolone" OR "Triamcinolone" OR "Betamethasone" OR "steroid" 54934  #13 "Captopril" 2547  #14 "Vincristine" OR "Sirolimus" OR "Interferon" 27102  #15 MeSH descriptor: [Lasers] explode all trees 3892  #16 MeSH descriptor: [Laser Therapy] explode all trees 6246  #17 MeSH descriptor: [Sclerotherapy] explode all trees 622  #18 "Laser" OR "Pulsed Lasers" OR "Pulsed Laser" OR "Laser Therapies" 28240  #19 "Bleomycin" OR "Polidocanol" OR "surgery" OR "excision" 359910  #20 #7 or #8 139  #21 #9 OR #10 OR #11 OR #12 OR #13 OR #14 OR #15 OR #16 OR #17 OR #18 OR #19 461009  #22 #20 AND #21 in Trials 124 |
|  |  |
|  |  |
|  |  |
| Embase | No. Search Hits  #1 'capillary hemangioma'/exp 5915  #2 angiomatous nevus':ab,ti OR 'capillary haemangioma':ab,ti OR 'capillary hemangioma of the skin':ab,ti OR 'capillary nevus':ab,ti OR 'cellular hemangioma':ab,ti OR 'haemangioma, capillary':ab,ti OR 'hemangioma simplex':ab,ti OR 'hemangioma, capillary':ab,ti OR 'infantile capillary haemangioma':ab,ti OR 'infantile capillary hemangioma':ab,ti OR 'infantile haemangioma':ab,ti OR 'infantile hemangioma':ab,ti OR 'juvenile capillary hemangioma':ab,ti OR 'juvenile haemangioma':ab,ti OR 'juvenile hemangioma':ab,ti OR 'plexiform hemangioma':ab,ti OR 'strawberry angioma':ab,ti OR 'strawberry birth mark':ab,ti OR 'strawberry birthmark':ab,ti OR 'strawberry haemangioma':ab,ti OR 'strawberry hemangioma':ab,ti OR 'strawberry naevi':ab,ti OR 'strawberry naevus':ab,ti OR 'strawberry nevi':ab,ti OR 'strawberry nevus':ab,ti OR 'capillary hemangioma':ab,ti 4319  #3 'infant'/exp 1440200  #4 'infant':ab,ti 278356  #5 #1 OR #2 7233  #6 #3 OR #4 1523181  #7 #5 AND #6 2574  #8 'beta adrenergic receptor blocking agent'/exp 389506  #9 'adrenergic beta antagonists':ab,ti OR 'adrenergic beta-antagonists':ab,ti OR 'antiadrenergics, beta blocking':ab,ti OR 'beta adrenergic antagonist':ab,ti OR 'beta adrenergic blocker':ab,ti OR 'beta adrenergic blockers':ab,ti OR 'beta adrenergic blocking agent':ab,ti OR 'beta adrenergic blocking drug':ab,ti OR 'beta adrenergic receptor antagonist':ab,ti OR 'beta adrenergic receptor blocker':ab,ti OR 'beta adrenoceptor antagonist':ab,ti OR 'beta adrenoceptor blocker':ab,ti OR 'beta adrenoceptor blocking agent':ab,ti OR 'beta adrenoceptor blocking drug':ab,ti OR 'beta adrenolytic':ab,ti OR 'beta adrenolytic agent':ab,ti OR 'beta antagonist':ab,ti OR 'beta antiadrenergic agent':ab,ti OR 'beta blocker':ab,ti OR 'beta blocking adrenergic agent':ab,ti OR 'beta blocking agent':ab,ti OR 'beta blocking drug':ab,ti OR 'beta receptor adrenergic blocking agent':ab,ti OR 'beta receptor blocker':ab,ti OR 'beta receptor blocking agent':ab,ti OR 'beta sympathicolytic agent':ab,ti OR 'beta sympathicolytics':ab,ti OR 'beta sympatholytic agent':ab,ti OR 'betasympatholytic agent':ab,ti OR 'beta adrenergic receptor blocking agent':ab,ti 22286  #10 'propranolol':ab,ti OR 'atenolol':ab,ti OR 'nadolol':ab,ti OR 'timolol':ab,ti OR 'carteolol':ab,ti 61281  #11 'captopril':ab,ti 15735  #12 'glucocorticoid'/exp 1049913  #13 'glucocorticoid drug':ab,ti OR 'glucocorticoid hormone':ab,ti OR 'glucocorticoid steroid':ab,ti OR 'glucocorticoids':ab,ti OR 'glucocorticoids, synthetic':ab,ti OR 'glucocorticoids, topical':ab,ti OR 'glucocorticoidsteroid':ab,ti OR 'glucocorticosteroid':ab,ti OR 'glucocortoid':ab,ti OR 'glycocorticoid':ab,ti OR 'glycocorticosteroid':ab,ti OR 'glucocorticoid':ab,ti 126186  #14 'corticosteroids':ab,ti OR 'prednisone':ab,ti OR 'prednisolone':ab,ti OR 'triamcinolone':ab,ti OR 'betamethasone':ab,ti OR 'steroid':ab,ti 496371  #15 'vincristine':ab,ti OR 'sirolimus':ab,ti OR 'interferon':ab,ti 300884  #16 'laser'/exp 238080  #17 'beam, laser':ab,ti OR 'laser adapter':ab,ti OR 'laser adaptor':ab,ti OR 'laser associate':ab,ti OR 'laser beam':ab,ti OR 'laser device':ab,ti OR 'laser holder':ab,ti OR 'laser irradiation':ab,ti OR 'laser radiation':ab,ti OR 'laser ray':ab,ti OR 'lasers':ab,ti OR 'radiation, laser':ab,ti OR 'laser':ab,ti 386859  #18 'sclerotherapy'/exp 17674  #19 'sclerosation':ab,ti OR 'sclerosing injection therapy':ab,ti OR 'sclerosing therapy':ab,ti OR 'sclerozation':ab,ti OR 'sclerotherapy':ab,ti 13510  #20 'bleomycin':ab,ti OR 'polidocanol':ab,ti OR 'surgery':ab,ti OR 'excision':ab,ti 2563131  #21 #8 OR #9 OR #10 OR #11 OR #12 OR #13 OR #14 OR #15 OR #16 OR #17 OR #18 OR #19 OR #20  #22 #7 AND #21 4825780  #23 #22 AND 'randomized controlled trial'/de 60 |
|  |  |
|  |  |
|  |  |
|  |  |
|  |  |
|  |  |
|  |  |
|  |  |
|  |  |
|  |  |

Supplementary Table3. Global inconsistency table for Marked Effective Rate and Recurrence Rate

| **Outcome** | **P-value for Global Inconsistency Analysis** |
| --- | --- |
| Marked Effective Rate | 0.0789 |
| Recurrence Rate | 0.6633 |

Supplementary Table4. Node-splitting analysis for the Marked Effective Rate outcome in IH

| **Side** | **Coef.** | **Std. Err.** | **Coef.** | **Std. Err.** | **Coef.** | **Std. Err.** | **P>z** | **tau** | |
| --- | --- | --- | --- | --- | --- | --- | --- | --- | --- |
| A E | 0.3339995 | 0.2143435 | -0.17023 | 5.776004 | 0.5042295 | 5.779819 | 0.93 | | 0.1253676 |
| A H | 0.4700036 | 0.4430918 | -0.685115 | 0.6673822 | 1.155119 | 0.8897918 | 0.194 | | 0.0682911 |
| B C | 1.003302 | 0.4534698 | -0.9651811 | 44.73291 | 1.968483 | 44.73926 | 0.965 | | 0.1248661 |
| B G | 0.305489 | 0.1007537 | 1.103054 | 0.362533 | -0.797565 | 0.3762723 | 0.054 | | 5.14E-09 |
| B I | 0.5877763 | 0.2981417 | -0.2094147 | 0.2295842 | 0.797191 | 0.3762934 | 0.054 | | 2.55E-07 |
| D E | -0.2076394 | 0.2345732 | 0.6773854 | 38.95677 | -0.8850247 | 38.95785 | 0.982 | | 0.124906 |
| E F | 0.0661819 | 0.1529908 | -0.676832 | 21.61179 | 0.7430139 | 21.61248 | 0.973 | | 0.1250333 |
| E G | 0.5930595 | 0.1796329 | -0.204481 | 0.3305573 | 0.7975405 | 0.3762153 | 0.054 | | 1.59E-09 |
| E H | -0.3184538 | 0.2953702 | 0.8366658 | 0.8790338 | -1.15512 | 0.8898102 | 0.194 | | 0.0683212 |
| E I | 0.0779458 | 0.1014893 | 0.8738786 | 0.3621829 | -0.7959328 | 0.3761337 | 0.054 | | 1.43E-08 |
| E K | 0.2006707 | 0.2903691 | 0.9208575 | 0.7296365 | -0.7201868 | 0.8106095 | 0.374 | | 0.1901057 |
| I J | 0.223143 | 0.1452914 | -0.9759742 | 27.60491 | 1.199117 | 27.60542 | 0.965 | | 0.1250336 |

Supplementary Table5. Node-splitting analysis for the Recurrence Rate outcome in IH

|  | **Coef.** | **Std. Err.** | **Coef.** | **Std. Err.** | **Coef.** | **Std. Err.** | **P>z** | **tau** |
| --- | --- | --- | --- | --- | --- | --- | --- | --- |
| A D | -0.6928879 | 1.169017 | 0.6898757 | 2.783452 | -1.382764 | 3.179885 | 0.664 | 0.0041468 |
| A E | -0.6937285 | 1.169013 | -2.07316 | 2.784512 | 1.379431 | 3.180896 | 0.665 | 0.0001352 |
| B D | 0.4436169 | 0.3540539 | -0.8231307 | 210.9882 | 1.266748 | 210.9884 | 0.995 | 9.82E-07 |
| C D | -0.4054651 | 0.8563488 | -0.8344351 | 167.3659 | 0.42897 | 167.3691 | 0.998 | 6.75E-09 |
| D E | -0.5107488 | 0.7008758 | -1.262707 | 131.1993 | 0.7519583 | 131.2012 | 0.995 | 2.96E-07 |

Supplementary Table6. Loop-specific inconsistency test table for the Marked Effective Rate outcome in IH

| **Loop** | **IF** | **seIF** | **z_value** | **p_value** | **CI_95** | **Loop_Heterog_tau2** |
| --- | --- | --- | --- | --- | --- | --- |
| Laser-OP-OCS-OP-ILL-TP | 0.797 | 0.376 | 2.119 | 0.054 | (0.00,1.53) | 0 |
| ILP-OP-OCS-OP-Laser | 0.577 | 0.553 | 1.045 | 0.296 | (0.00,1.66) | 0 |
| OP-OCS-TP-TT | 0.224 | 0.349 | 0.641 | 0.522 | (0.00,0.91) | 0 |

Supplementary Table7. Loop-specific inconsistency test table for the Recurrence Rate outcome in IH

| **Loop** | **IF** | **seIF** | **z_value** | **p_value** | **CI_95** | **Loop_Heterog_tau2** |
| --- | --- | --- | --- | --- | --- | --- |
| ILP-OP-TP | 0.788 | 2.035 | 0.387 | 0.698 | (0.00,4.78) | 0 |

Supplementary Table8. SUCRA ranking table for the Excellent Response Rate outcome in IH

| **Treatm~t** | **SUCRA** | **PrBest** | **MeanRank** |
| --- | --- | --- | --- |
| TT-ILL | 95.2 | 76.9 | 1.3 |
| TT | 73.5 | 0.2 | 2.9 |
| Laser-TP | 66 | 18.1 | 3.4 |
| OP-Laser | 55 | 1 | 4.1 |
| OP-OCS | 46.3 | 3.5 | 4.8 |
| OP-ILB | 32.1 | 0.3 | 5.8 |
| Laser | 26.4 | 0 | 6.2 |
| OP | 5.4 | 0 | 7.6 |

Supplementary Table9. SUCRA ranking table for the Marked Effective Rate outcome in IH

| **Treatm~t** | **SUCRA** | **PrBest** | **MeanRank** |
| --- | --- | --- | --- |
| Laser-TP | 94.6 | 82.2 | 1.5 |
| OP-Laser | 76.4 | 3.4 | 3.4 |
| TT-ILL | 74.7 | 6.1 | 3.5 |
| TT-OP | 67.4 | 5.3 | 4.3 |
| OP-OCS | 57 | 2.6 | 5.3 |
| TT | 49.4 | 0 | 6.1 |
| OP-ILL | 40.7 | 0.1 | 6.9 |
| Laser | 35 | 0 | 7.5 |
| OP | 29.9 | 0 | 8 |
| TP | 17.1 | 0.3 | 9.3 |
| ILP | 7.7 | 0 | 10.2 |

Supplementary Table10. SUCRA ranking table for the Total Effective Rate outcome in IH

| **Treatm~t** | **SUCRA** | **PrBest** | **MeanRank** |
| --- | --- | --- | --- |
| Laser-TP | 83.2 | 67.2 | 1.8 |
| OP-Laser | 70.1 | 15.2 | 2.5 |
| TT-ILL | 61.6 | 15.5 | 2.9 |
| TT | 44.7 | 2 | 3.8 |
| OP | 24.9 | 0.1 | 4.8 |
| Laser | 15.5 | 0 | 5.2 |

Supplementary Table11. SUCRA ranking table for the VAS (Color) outcome in IH

| **Treatm~t** | **SUCRA** | **PrBest** | **MeanRank** |
| --- | --- | --- | --- |
| ON | 90.4 | 62.7 | 1.4 |
| OP-TT | 81.4 | 35.7 | 1.7 |
| OP-OCS | 35.8 | 1.1 | 3.6 |
| OP | 34.4 | 0 | 3.6 |
| OCS | 8 | 0.4 | 4.7 |

Supplementary Table12.SUCRA ranking table for the VAS (Size) outcome in IH

| **Treatm~t** | **SUCRA** | **PrBest** | **MeanRank** |
| --- | --- | --- | --- |
| OP-Laser | 99.9 | 99.2 | 1 |
| Laser | 81.3 | 0.3 | 2.1 |
| ON | 52 | 0 | 3.9 |
| OP-TT | 50.1 | 0.3 | 4 |
| OP-OCS | 36.7 | 0.2 | 4.8 |
| OP | 29.6 | 0 | 5.2 |
| OCS | 0.5 | 0 | 7 |

Supplementary Table13.SUCRA ranking table for the HAS outcome in IH

| **Treatm~t** | **SUCRA** | **PrBest** | **MeanRank** |
| --- | --- | --- | --- |
| OP-TT | 98.7 | 96.1 | 1 |
| ON | 63 | 3.9 | 2.1 |
| OP | 31.1 | 0 | 3.1 |
| OA | 7.2 | 0 | 3.8 |

Supplementary Table14.SUCRA ranking table for the Healing Time outcome in IH

| **Treatm~t** | **SUCRA** | **PrBest** | **MeanRank** |
| --- | --- | --- | --- |
| OP-Laser | 100 | 100 | 1 |
| Laser | 66.7 | 0 | 2 |
| OA | 31.4 | 0 | 3.1 |
| OP | 1.9 | 0 | 3.9 |

Supplementary Table15.SUCRA ranking table for the TEAE outcome in IH

| **Treatm~t** | **SUCRA** | **PrBest** | **MeanRank** |
| --- | --- | --- | --- |
| TT | 85.5 | 51.6 | 2.2 |
| OP-Laser | 77 | 10.9 | 2.8 |
| TP | 74.7 | 34.1 | 3 |
| OA | 43.4 | 0.5 | 5.5 |
| ON | 42.2 | 0.5 | 5.6 |
| Laser | 41.4 | 0.1 | 5.7 |
| WW | 35.2 | 2.4 | 6.2 |
| OP | 25.4 | 0 | 7 |
| OCS | 25.2 | 0 | 7 |

Supplementary Table16.SUCRA ranking table for the Recurrence Rate outcome in IH

| **Treatm~t** | **SUCRA** | **PrBest** | **MeanRank** |
| --- | --- | --- | --- |
| OA | 74.5 | 35.9 | 2 |
| TP | 73.3 | 43.2 | 2.1 |
| OP | 42.4 | 1.2 | 3.3 |
| ILP | 30.5 | 10.4 | 3.8 |
| OP-OCS | 29.4 | 9.3 | 3.8 |

Supplementary Table17.SUCRA ranking table for the Marked Effective Rate outcome in Superficial IH

| **Treatm~t** | **SUCRA** | **PrBest** | **MeanRank** |
| --- | --- | --- | --- |
| OP-Laser | 96.3 | 86.2 | 1.1 |
| TT-OP | 59.5 | 11.5 | 2.6 |
| Laser | 55.7 | 2.1 | 2.8 |
| TT | 28.4 | 0.2 | 3.9 |
| OP | 10.2 | 0 | 4.6 |

Supplementary Table18.Sensitivity analysis for the Excellent Response Rate outcome in IH

| **dropped_id** | **comparison** | **eff** | **lci** | **uci** | **connected** |
| --- | --- | --- | --- | --- | --- |
| Ehsani | LaservsOP | 1.5563683 | 0.53472 | 4.530001 | 1 |
| Ehsani | OP-ILBvsOP | 1.5538462 | 1.278524 | 1.888458 | 1 |
| Ehsani | OP-LaservsOP | 2.3987313 | 0.9154077 | 6.285628 | 1 |
| Ehsani | OP-OCSvsOP | 2 | 1.031018 | 3.879662 | 1 |
| Ehsani | TTvsOP | 4.6662218 | 0.9329391 | 23.33874 | 1 |
| Ehsani | TT-ILLvsOP | 7.9711438 | 1.521601 | 41.75807 | 1 |
| Aly | LaservsOP | 1.5561127 | 0.5346817 | 4.528838 | 1 |
| Aly | Laser-TPvsOP | 3.5005012 | 0.6160247 | 19.89126 | 1 |
| Aly | OP-ILBvsOP | 1.5538462 | 1.278524 | 1.888458 | 1 |
| Aly | OP-LaservsOP | 2.398411 | 0.9153476 | 6.284362 | 1 |
| Aly | TTvsOP | 4.6654558 | 0.9328433 | 23.33348 | 1 |
| Aly | TT-ILLvsOP | 7.9698353 | 1.521442 | 41.74872 | 1 |
| Guo | LaservsOP | 1.5561127 | 0.5346819 | 4.528837 | 1 |
| Guo | Laser-TPvsOP | 3.5005012 | 0.6160248 | 19.89126 | 1 |
| Guo | OP-LaservsOP | 2.398411 | 0.9153477 | 6.284361 | 1 |
| Guo | OP-OCSvsOP | 2 | 1.031018 | 3.879662 | 1 |
| Guo | TTvsOP | 4.6654558 | 0.9328436 | 23.33347 | 1 |
| Guo | TT-ILLvsOP | 7.9698353 | 1.521443 | 41.74871 | 1 |
| Zhang | LaservsOP | 1.5567646 | 0.5348408 | 4.531284 | 1 |
| Zhang | Laser-TPvsOP | 3.5019672 | 0.6162368 | 19.90107 | 1 |
| Zhang | OP-ILBvsOP | 1.5538462 | 1.278524 | 1.888458 | 1 |
| Zhang | OP-LaservsOP | 2.3992278 | 0.9155779 | 6.287061 | 1 |
| Zhang | OP-OCSvsOP | 2 | 1.031018 | 3.879662 | 1 |
| Zhang | TTvsOP | 4.669896 | 0.9333434 | 23.36539 | 1 |

Supplementary Table19.Sensitivity analysis for the Marked Effective Rate outcome in IH

| **dropped_id** | **comparison** | **eff** | **lci** | **uci** | **connected** |
| --- | --- | --- | --- | --- | --- |
| Gong | ILPvsOP | -0.8263679 | 0.1059315 | 0.69752412 | 1 |
| Gong | LaservsOP | -0.4879569 | 0.5113029 | 1.0117414 | 1 |
| Gong | Laser-TPvsOP | -0.0430882 | 2.072504 | 2.758557 | 1 |
| Gong | OP-OCSvsOP | -0.3319737 | 0.7472525 | 1.2307692 | 1 |
| Gong | OP-ILLvsOP | -0.3456371 | 0.4780009 | 1.0684211 | 1 |
| Gong | OP-LaservsOP | -0.0485651 | 0.8436206 | 1.4881411 | 1 |
| Gong | TPvsOP | -0.8833967 | 0.4338569 | 0.79869995 | 1 |
| Gong | TTvsOP | -0.1780418 | 0.6638697 | 1.2749589 | 1 |
| Gong | TT-ILLvsOP | -0.1152586 | 1.047288 | 1.5936301 | 1 |
| Zhao | ILPvsOP | -0.8916004 | 0.1372995 | 0.68581287 | 1 |
| Zhao | LaservsOP | -0.6332246 | 0.5987164 | 0.98289389 | 1 |
| Zhao | Laser-TPvsOP | -0.1622083 | 2.13377 | 2.6799043 | 1 |
| Zhao | OP-OCSvsOP | -0.3927351 | 0.8080138 | 1.2307692 | 1 |
| Zhao | OP-ILLvsOP | -0.4225533 | 0.5549171 | 1.0684211 | 1 |
| Zhao | OP-LaservsOP | -0.1480055 | 0.9074308 | 1.4618645 | 1 |
| Zhao | TPvsOP | -0.9229568 | 0.4856867 | 0.80361494 | 1 |
| Zhao | TTvsOP | -0.3789586 | 0.8151688 | 1.2437178 | 1 |
| Zhao | TT-ILLvsOP | -0.3245886 | 1.206974 | 1.5545603 | 1 |
| Zhao | TT-OPvsOP | -0.2471813 | 0.9502767 | 1.4212655 | 1 |
| Ehsani | ILPvsOP | -0.7542246 | 0.0861626 | 0.71603156 | 1 |
| Ehsani | LaservsOP | -0.4783686 | 0.4910515 | 1.0063616 | 1 |
| Ehsani | OP-OCSvsOP | -0.2526939 | 0.6679726 | 1.2307692 | 1 |
| Ehsani | OP-ILLvsOP | -0.2343498 | 0.3667136 | 1.0684211 | 1 |
| Ehsani | OP-LaservsOP | -0.0099916 | 0.787006 | 1.4747776 | 1 |
| Ehsani | TPvsOP | -0.8378137 | 0.3692976 | 0.79115764 | 1 |
| Ehsani | TTvsOP | -0.127041 | 0.4238441 | 1.1599786 | 1 |
| Ehsani | TT-ILLvsOP | -0.0251372 | 0.7681928 | 1.4499481 | 1 |
| Ehsani | TT-OPvsOP | -0.1230178 | 0.7528204 | 1.370124 | 1 |
| Yang | ILPvsOP | -0.6220982 | 0.0093826 | 0.73612319 | 1 |
| Yang | LaservsOP | -1.126832 | 0.1073638 | 0.60065543 | 1 |
| Yang | Laser-TPvsOP | -0.5602047 | 1.547241 | 1.6380693 | 1 |
| Yang | OP-OCSvsOP | -0.1815234 | 0.5968022 | 1.2307692 | 1 |
| Yang | OP-ILLvsOP | -0.1066194 | 0.2389832 | 1.0684211 | 1 |
| Yang | OP-LaservsOP | -0.8521379 | 0.4436756 | 0.81527388 | 1 |
| Yang | TPvsOP | -0.7967834 | 0.3082142 | 0.7832647 | 1 |
| Yang | TTvsOP | -0.1209582 | 0.276855 | 1.0810669 | 1 |
| Yang | TT-ILLvsOP | 0.0549189 | 0.5472557 | 1.3513273 | 1 |
| Yang | TT-OPvsOP | -0.0885495 | 0.6525767 | 1.3257968 | 1 |
| Zaher | ILPvsOP | -0.5822509 | 0.1596327 | 0.80952381 | 1 |
| Zaher | LaservsOP | -0.4742169 | 0.5413002 | 1.0341105 | 1 |
| Zaher | Laser-TPvsOP | 0.0337263 | 2.039451 | 2.8195825 | 1 |
| Zaher | OP-OCSvsOP | -0.2043056 | 0.6195843 | 1.2307692 | 1 |
| Zaher | OP-ILLvsOP | -0.1531609 | 0.2855247 | 1.0684211 | 1 |
| Zaher | OP-LaservsOP | 0.0218824 | 0.7846138 | 1.4966782 | 1 |
| Zaher | TTvsOP | -0.1073957 | 0.3902124 | 1.1518949 | 1 |
| Zaher | TT-ILLvsOP | 0.046475 | 0.68261 | 1.4398551 | 1 |
| Zaher | TT-OPvsOP | -0.0890102 | 0.7081726 | 1.3628542 | 1 |
| Tawfik | ILPvsOP | -0.6220983 | 0.0093826 | 0.73612318 | 1 |
| Tawfik | LaservsOP | -0.1162126 | 0.6910686 | 1.3329947 | 1 |
| Tawfik | Laser-TPvsOP | 0.3456037 | 2.235165 | 3.6341824 | 1 |
| Tawfik | OP-OCSvsOP | -0.1815234 | 0.5968022 | 1.2307692 | 1 |
| Tawfik | OP-ILLvsOP | -0.1066194 | 0.2389832 | 1.0684211 | 1 |
| Tawfik | OP-LaservsOP | 0.2408973 | 0.9449914 | 1.8093078 | 1 |
| Tawfik | TPvsOP | -0.7967834 | 0.3082143 | 0.78326471 | 1 |
| Tawfik | TTvsOP | -0.1209786 | 0.2768488 | 1.0810525 | 1 |
| Tawfik | TT-ILLvsOP | 0.0548999 | 0.5472481 | 1.3513094 | 1 |
| Tawfik | TT-OPvsOP | -0.0885556 | 0.6525719 | 1.3257895 | 1 |
| Mehta | ILPvsOP | -1.578137 | 0.0012224 | 0.45454545 | 1 |
| Mehta | LaservsOP | -0.4742098 | 0.541293 | 1.0341104 | 1 |
| Mehta | Laser-TPvsOP | 0.0337298 | 2.039448 | 2.8195824 | 1 |
| Mehta | OP-OCSvsOP | -0.2043057 | 0.6195844 | 1.2307692 | 1 |
| Mehta | OP-ILLvsOP | -0.153161 | 0.2855248 | 1.0684211 | 1 |
| Mehta | OP-LaservsOP | 0.021885 | 0.7846111 | 1.4966781 | 1 |
| Mehta | TPvsOP | -0.897655 | 0.2607475 | 0.72727273 | 1 |
| Mehta | TTvsOP | -0.107394 | 0.3902107 | 1.1518949 | 1 |
| Mehta | TT-ILLvsOP | 0.0464762 | 0.6826088 | 1.4398552 | 1 |
| Mehta | TT-OPvsOP | -0.0890098 | 0.7081722 | 1.3628542 | 1 |
| Aly | ILPvsOP | -0.7542294 | 0.0861565 | 0.71602767 | 1 |
| Aly | LaservsOP | -0.4784667 | 0.4910217 | 1.0062972 | 1 |
| Aly | Laser-TPvsOP | -0.0031408 | 2.021796 | 2.7437559 | 1 |
| Aly | OP-ILLvsOP | -0.2343728 | 0.3667366 | 1.0684211 | 1 |
| Aly | OP-LaservsOP | -0.0100568 | 0.786981 | 1.4747111 | 1 |
| Aly | TPvsOP | -0.8378209 | 0.3693088 | 0.7911592 | 1 |
| Aly | TTvsOP | -0.1270516 | 0.4238384 | 1.1599692 | 1 |
| Aly | TT-ILLvsOP | -0.0251645 | 0.7682038 | 1.4499363 | 1 |
| Aly | TT-OPvsOP | -0.1230313 | 0.7528282 | 1.3701202 | 1 |
| Ma | ILPvsOP | -0.7542285 | 0.0861556 | 0.71602769 | 1 |
| Ma | LaservsOP | -0.4784654 | 0.4910204 | 1.0062972 | 1 |
| Ma | Laser-TPvsOP | -0.0031401 | 2.021796 | 2.743756 | 1 |
| Ma | OP-OCSvsOP | -0.2527088 | 0.6679875 | 1.2307692 | 1 |
| Ma | OP-LaservsOP | -0.0100563 | 0.7869805 | 1.4747111 | 1 |
| Ma | TPvsOP | -0.8378208 | 0.3693086 | 0.79115919 | 1 |
| Ma | TTvsOP | -0.1270513 | 0.4238382 | 1.1599692 |  |
| Ma | TT-ILLvsOP | -0.0251641 | 0.7682034 | 1.4499363 |  |
| Ma | TT-OPvsOP | -0.1230311 | 0.7528281 | 1.3701202 |  |
| Zhang | ILPvsOP | -0.7543089 | 0.0861792 | 0.71600732 |  |
| Zhang | LaservsOP | -0.4784987 | 0.4909947 | 1.0062676 |  |
| Zhang | Laser-TPvsOP | -0.0032073 | 2.021804 | 2.7436752 |  |
| Zhang | OP-OCSvsOP | -0.2527879 | 0.6680666 | 1.2307692 |  |
| Zhang | OP-ILLvsOP | -0.2344938 | 0.3668576 | 1.0684211 |  |
| Zhang | OP-LaservsOP | -0.0101115 | 0.7870072 | 1.4746901 |  |
| Zhang | TPvsOP | -0.837864 | 0.3693725 | 0.79116734 |  |
| Zhang | TTvsOP | -0.1271112 | 0.4239672 | 1.1600093 |  |
| Zhang | TT-OPvsOP | -0.1230885 | 0.7529224 | 1.3701455 |  |

Supplementary Table20.Sensitivity analysis for the Total Effective Rate outcome in IH

| **dropped_id** | **comparison** | **eff** | **lci** | **uci** | **connected** |
| --- | --- | --- | --- | --- | --- |
| Ehsani | LaservsOP | 0.96772405 | 0.8119646 | 1.153363 | 1 |
| Ehsani | OP-LaservsOP | 1.2050902 | 1.045553 | 1.38897 | 1 |
| Ehsani | TTvsOP | 1.1203568 | 0.7593409 | 1.653012 | 1 |
| Ehsani | TT-ILLvsOP | 1.1514749 | 0.7776204 | 1.705066 | 1 |
| Zhang | LaservsOP | 0.96775399 | 0.8119714 | 1.153425 | 1 |
| Zhang | Laser-TPvsOP | 1.4211165 | 0.8583284 | 2.352913 | 1 |
| Zhang | OP-LaservsOP | 1.2051146 | 1.045562 | 1.389015 | 1 |
| Zhang | TTvsOP | 1.1205552 | 0.7592639 | 1.653765 | 1 |

Supplementary Table21.Sensitivity analysis for the TEAE outcome in IH

| **dropped_id** | **comparison** | **eff** | **lci** | **uci** | **connected** |
| --- | --- | --- | --- | --- | --- |
| Ji | LaservsOP | 0.70411914 | 0.149113 | 3.324886 | 1 |
| Ji | OAvsOP | 1.1666667 | 0.7374817 | 1.84562 | 1 |
| Ji | OCSvsOP | 1.0082606 | 0.8608541 | 1.180908 | 1 |
| Ji | ONvsOP | 0.81680672 | 0.6707717 | 0.9946353 | 1 |
| Ji | OP-LaservsOP | 0.30004599 | 0.088008 | 1.022948 | 1 |
| Ji | WWvsOP | 0.99431728 | 0.0361646 | 27.338 | 1 |
| Ji | TPvsOP | 0.24 | 0.0288493 | 1.996583 | 1 |
| Ji | TTvsOP | 0.14084533 | 0.0104583 | 1.896811 | 1 |
| Ashraf | LaservsOP | 0.70411914 | 0.149113 | 3.324886 | 1 |
| Ashraf | OAvsOP | 0.63407181 | 0.526722 | 0.7633003 | 1 |
| Ashraf | OCSvsOP | 1.0082606 | 0.8608541 | 1.180908 | 1 |
| Ashraf | ONvsOP | 0.81680672 | 0.6707717 | 0.9946353 | 1 |
| Ashraf | OP-LaservsOP | 0.30004599 | 0.088008 | 1.022948 | 1 |
| Ashraf | WWvsOP | 0.99431728 | 0.0361646 | 27.338 | 1 |
| Ashraf | TPvsOP | 0.24 | 0.0288493 | 1.996583 | 1 |
| Ashraf | TTvsOP | 0.14084533 | 0.0104583 | 1.896811 | 1 |
| Liu | LaservsOP | 0.37516412 | 0.0574379 | 2.450439 | 1 |
| Liu | OAvsOP | 0.7955736 | 0.5179703 | 1.221957 | 1 |
| Liu | OCSvsOP | 1.0252711 | 0.6963366 | 1.509587 | 1 |
| Liu | ONvsOP | 0.81680673 | 0.4903288 | 1.360665 | 1 |
| Liu | OP-LaservsOP | 0.30006763 | 0.0806604 | 1.116292 | 1 |
| Liu | WWvsOP | 0.52992434 | 0.0153634 | 18.2785 | 1 |
| Liu | TPvsOP | 0.24 | 0.0273963 | 2.102474 | 1 |
| Liu | TTvsOP | 0.07505568 | 0.0043627 | 1.29124 | 1 |
| Sun | LaservsOP | 1.649963 | 0.2190859 | 12.42607 | 1 |
| Sun | OAvsOP | 0.79557374 | 0.5179704 | 1.221957 | 1 |
| Sun | OCSvsOP | 1.0252711 | 0.6963369 | 1.509587 | 1 |
| Sun | ONvsOP | 0.81680672 | 0.4903291 | 1.360664 | 1 |
| Sun | OP-LaservsOP | 0.2999972 | 0.0806423 | 1.116018 | 1 |
| Sun | WWvsOP | 2.3292318 | 0.0624975 | 86.80864 | 1 |
| Sun | TPvsOP | 0.24 | 0.0273963 | 2.102474 | 1 |
| Sun | TTvsOP | 0.32998274 | 0.0174282 | 6.247844 | 1 |
| Bauman | LaservsOP | 0.72766583 | 0.1094983 | 4.835671 | 1 |
| Bauman | OAvsOP | 0.83481767 | 0.4257162 | 1.637054 | 1 |
| Bauman | OCSvsOP | 0.99609376 | 0.4056038 | 2.446237 | 1 |
| Bauman | ONvsOP | 0.81680673 | 0.3310028 | 2.015612 | 1 |
| Bauman | OP-LaservsOP | 0.30006539 | 0.0662669 | 1.358737 | 1 |
| Bauman | WWvsOP | 1.0276013 | 0.0253307 | 41.68713 | 1 |
| Bauman | TPvsOP | 0.24 | 0.0241914 | 2.38101 | 1 |
| Bauman | TTvsOP | 0.14555911 | 0.0076026 | 2.786877 | 1 |
| Kim | LaservsOP | 0.72766583 | 0.1094983 | 4.835671 | 1 |
| Kim | OAvsOP | 0.83481767 | 0.4257161 | 1.637055 | 1 |
| Kim | OCSvsOP | 1.0694444 | 0.4091169 | 2.795561 | 1 |
| Kim | ONvsOP | 0.81680673 | 0.3310028 | 2.015612 | 1 |
| Kim | OP-LaservsOP | 0.30006539 | 0.0662669 | 1.358737 | 1 |
| Kim | WWvsOP | 1.0276013 | 0.0253307 | 41.68713 | 1 |
| Kim | TPvsOP | 0.24 | 0.0241914 | 2.38101 | 1 |
| Kim | TTvsOP | 0.14555911 | 0.0076026 | 2.786877 | 1 |
| Abdel Wahab | LaservsOP | 0.71368033 | 0.1340442 | 3.799789 | 1 |
| Abdel Wahab | OAvsOP | 0.80116474 | 0.5116671 | 1.254458 | 1 |
| Abdel Wahab | OCSvsOP | 1.0259453 | 0.6816445 | 1.544153 | 1 |
| Abdel Wahab | ONvsOP | 0.81680673 | 0.4747725 | 1.405248 | 1 |
| Abdel Wahab | OP-LaservsOP | 0.30005243 | 0.0796296 | 1.130628 | 1 |
| Abdel Wahab | WWvsOP | 1.007829 | 0.0320991 | 31.64326 | 1 |
| Abdel Wahab | TTvsOP | 0.14275902 | 0.0093952 | 2.169212 | 1 |
| Pope | LaservsOP | 0.71368034 | 0.1340442 | 3.799789 | 1 |
| Pope | OAvsOP | 0.80116474 | 0.5116673 | 1.254458 | 1 |
| Pope | OCSvsOP | 1.0259453 | 0.6816444 | 1.544154 | 1 |
| Pope | OP-LaservsOP | 0.30005243 | 0.0796296 | 1.130628 | 1 |
| Pope | WWvsOP | 1.007829 | 0.0320991 | 31.64326 | 1 |
| Pope | TPvsOP | 0.24 | 0.0271832 | 2.118952 | 1 |
| Pope | TTvsOP | 0.14275902 | 0.0093952 | 2.169212 | 1 |
| Cheng | LaservsOP | 0.71361935 | 0.134018 | 3.799881 | 1 |
| Cheng | OAvsOP | 0.80116651 | 0.5116641 | 1.254471 | 1 |
| Cheng | OCSvsOP | 1.0259455 | 0.6816388 | 1.544167 | 1 |
| Cheng | ONvsOP | 0.81680672 | 0.4747666 | 1.405266 | 1 |
| Cheng | OP-LaservsOP | 0.30003616 | 0.0796207 | 1.130631 | 1 |
| Cheng | TPvsOP | 0.24 | 0.0271832 | 2.118958 | 1 |
| Cheng | TTvsOP | 0.14272656 | 0.0093888 | 2.169706 | 1 |

Supplementary Table22.Meta-regression analysis of the Excellent Response Rate outcome with mean age as a moderator in IH

| **Intervention** | **Covariate** | **Coefficient** | **Standard** Error | **Z-statistic** | **P>z** | **lower confidence interval** | **upper confidence interval** |
| --- | --- | --- | --- | --- | --- | --- | --- |
| LaservsOP | Mean age | -0.1949595 | 8.603425 | -0.02 | 0.982 | -17.05736 | 16.66744 |
|  | Intercept | 1.04242 | 30.21383 | 0.03 | 0.972 | -58.17559 | 60.26043 |
| Laser-TPvsOP | Intercept | 0.2390261 | 41.03045 | 0.01 | 0.995 | -80.17919 | 80.65724 |
| OP-ILBvsOP | Intercept | 0.4407346 | 0.0995403 | 4.43 | 0 | 0.2456393 | 0.63583 |
| OP-LaservsOP | Mean age | 0.3555317 | 8.846597 | 0.04 | 0.968 | -16.98348 | 17.69454 |
|  | Intercept | -0.3729601 | 31.06111 | -0.01 | 0.99 | -61.25163 | 60.50571 |
| OP-OCSvsOP | Intercept | 0.6931478 | 0.3380622 | 2.05 | 0.04 | 0.0305581 | 1.355738 |
| TTvsOP | Mean age | -2.759261 | 119.5153 | -0.02 | 0.982 | -237.0049 | 231.4864 |
|  | Intercept | 14.32107 | 568.6339 | 0.03 | 0.98 | -1100.181 | 1128.823 |
| TT-ILLvsOP | Intercept | 0.2324836 | 65.76647 | 0 | 0.997 | -128.6674 | 129.1324 |

Supplementary Table23.Meta-regression analysis of the Marked Effective Rate outcome with mean age as a moderator in IH

| **Intervention** | **Covariate** | **Coefficient** | **Standard Error** | **Z-statistic** | **P>z** | **lower confidence interval** | **upper confidence interval** |
| --- | --- | --- | --- | --- | --- | --- | --- |
| ILPvsOP | Mean age | 0.1288277 | 0.0969487 | 1.33 | 0.184 | -0.0611883 | 0.3188437 |
|  | Intercept | -1.924718 | 1.228497 | -1.57 | 0.117 | -4.332528 | 0.4830924 |
| LaservsOP | Mean age | -0.9763316 | 0.5699303 | -1.71 | 0.087 | -2.093374 | 0.1407112 |
|  | Intercept | 3.705735 | 2.083724 | 1.78 | 0.075 | -0.3782883 | 7.789758 |
| Laser-TPvsOP | Intercept | -3.373602 | 2.68973 | -1.25 | 0.21 | -8.645376 | 1.898171 |
| OP-OCSvsOP | Intercept | 0.2076394 | 0.1985525 | 1.05 | 0.296 | -0.1815163 | 0.596795 |
| OP-ILLvsOP | Intercept | 0.0661819 | 0.088164 | 0.75 | 0.453 | -0.1066162 | 0.2389801 |
| OP-LaservsOP | Mean age | -0.9072769 | 1.083601 | -0.84 | 0.402 | -3.031095 | 1.216541 |
|  | Intercept | 3.776841 | 3.831277 | 0.99 | 0.324 | -3.732324 | 11.28601 |
| TPvsOP | Intercept | -0.3184537 | 0.28736 | -1.11 | 0.268 | -0.8816689 | 0.2447615 |
| TTvsOP | Mean age | 0.3118796 | 0.4356032 | 0.72 | 0.474 | -0.541887 | 1.165646 |
|  | Intercept | -1.828067 | 2.664291 | -0.69 | 0.493 | -7.049981 | 3.393847 |
| TT-ILLvsOP | Intercept | 0.0480403 | 0.3752892 | 0.13 | 0.898 | -0.6875131 | 0.7835937 |
| TT-OPvsOP | Intercept | 0.2027712 | 0.2191217 | 0.93 | 0.355 | -0.2266994 | 0.6322418 |

Supplementary Table24.Meta-regression analysis of the TEAE outcome with mean age as a moderator in IH

| **Intervention** | **Covariate** | **Coefficient** | **Standard Error** | **Z-statistic** | **P>z** | **lower confidence interval** | **upper confidence interval** |
| --- | --- | --- | --- | --- | --- | --- | --- |
| LaservsOP | Mean age | 0.8938505 | 74.81092 | 0.01 | 0.99 | -145.7329 | 147.5206 |
|  | Intercept | -4.179296 | 262.5942 | -0.02 | 0.987 | -518.8545 | 510.4959 |
| OAvsOP | Mean age | 0.3586726 | 0.1490027 | 2.41 | 0.016 | 0.0666326 | 0.6507126 |
|  | Intercept | -1.298474 | 0.3959415 | -3.28 | 0.001 | -2.074505 | -0.5224431 |
| OCSvsOP | Mean age | -0.4179605 | 1.260862 | -0.33 | 0.74 | -2.889205 | 2.053284 |
|  | Intercept | 1.375355 | 4.124927 | 0.33 | 0.739 | -6.709354 | 9.460064 |
| ONvsOP | Intercept | -0.202353 | 0.100602 | -2.01 | 0.044 | -0.3995293 | -0.0051766 |
| OP-LaservsOP | Mean age | 7.064401 | 74.93591 | 0.09 | 0.925 | -139.8073 | 153.9361 |
|  | Intercept | -25.99968 | 263.0287 | -0.1 | 0.921 | -541.5264 | 489.5271 |
| WWvsOP | Intercept | 0.6980358 | 76.40324 | 0.01 | 0.993 | -149.0496 | 150.4456 |
| TPvsOP | Intercept | -1.427116 | 1.080895 | -1.32 | 0.187 | -3.545632 | 0.6913989 |
| TTvsOP | Mean age | -0.1467654 | 61.59528 | 0 | 0.998 | -120.8713 | 120.5778 |
|  | Intercept | -0.8453511 | 228.7446 | 0 | 0.997 | -449.1766 | 447.4859 |

SMI

Supplementary Figure1.Comparison-adjusted funnel plot for the Marked Effective Rate outcome in IH

Supplementary Figure2.Comparison-adjusted funnel plot for the TEAE outcome in IH

Supplementary Table25.GRADE certainty of evidence for the Excellent Response Rate outcome in IH

| **Comparison** | **Number of studies** | **Within-study bias** | **Reporting bias** | **Indirectness** | **Imprecision** | **Heterogeneity** | **Incoherence** | **Confidence rating** | **Reason**  **(s) for downgrading** |
| --- | --- | --- | --- | --- | --- | --- | --- | --- | --- |
| Laser:Laser-TP | 1 | Some concerns | Low risk | No concerns | Major concerns | No concerns | No concerns | Very low | [] |
| Laser:OP-Laser | 1 | Some concerns | Low risk | No concerns | Major concerns | No concerns | No concerns | Very low | [] |
| Laser:TT | 2 | Some concerns | Low risk | No concerns | Some concerns | No concerns | No concerns | Low | [] |
| OP:OP-ILB | 1 | Some concerns | Low risk | No concerns | Major concerns | No concerns | No concerns | Very low | [] |
| OP:OP-Laser | 1 | No concerns | Low risk | No concerns | No concerns | No concerns | No concerns | High | [] |
| OP:OP-OCS | 1 | Some concerns | Low risk | No concerns | Some concerns | No concerns | No concerns | Low | [] |
| TT:TT-ILL | 1 | Some concerns | Low risk | No concerns | No concerns | No concerns | No concerns | Moderate | [] |
| Laser:OP | 1 | Some concerns | Low risk | No concerns | No concerns | No concerns | No concerns | Moderate | [] |
| Laser:OP-ILB | 0 | Some concerns | Low risk | No concerns | Major concerns | No concerns | No concerns | Very low | [] |
| Laser:OP-OCS | 0 | Some concerns | Low risk | No concerns | Major concerns | No concerns | No concerns | Very low | [] |
| Laser:TT-ILL | 0 | Some concerns | Low risk | No concerns | Major concerns | No concerns | No concerns | Very low | [] |
| Laser-TP:OP | 0 | Some concerns | Low risk | No concerns | No concerns | No concerns | No concerns | Moderate | [] |
| Laser-TP:OP-ILB | 0 | Some concerns | Low risk | No concerns | Major concerns | No concerns | No concerns | Very low | [] |
| Laser-TP:OP-Laser | 0 | Some concerns | Low risk | No concerns | Major concerns | No concerns | No concerns | Very low | [] |
| Laser-TP:OP-OCS | 0 | Some concerns | Low risk | No concerns | Major concerns | No concerns | No concerns | Very low | [] |
| Laser-TP:TT | 0 | Some concerns | Low risk | No concerns | Major concerns | No concerns | No concerns | Very low | [] |
| Laser-TP:TT-ILL | 0 | Some concerns | Low risk | No concerns | Major concerns | No concerns | No concerns | Very low | [] |
| OP:TT | 0 | Some concerns | Low risk | No concerns | Major concerns | No concerns | No concerns | Very low | [] |
| OP:TT-ILL | 0 | Some concerns | Low risk | No concerns | Some concerns | No concerns | No concerns | Low | [] |
| OP-ILB:OP-Laser | 0 | Some concerns | Low risk | No concerns | No concerns | No concerns | No concerns | Moderate | [] |
| OP-ILB:OP-OCS | 0 | No concerns | Low risk | No concerns | Major concerns | No concerns | No concerns | Low | [] |
| OP-ILB:TT | 0 | No concerns | Low risk | No concerns | Major concerns | No concerns | No concerns | Low | [] |
| OP-ILB:TT-ILL | 0 | Some concerns | Low risk | No concerns | Major concerns | No concerns | No concerns | Very low | [] |
| OP-Laser:OP-OCS | 0 | Some concerns | Low risk | No concerns | Some concerns | No concerns | No concerns | Low | [] |
| OP-Laser:TT | 0 | Some concerns | Low risk | No concerns | Major concerns | No concerns | No concerns | Very low | [] |
| OP-Laser:TT-ILL | 0 | Some concerns | Low risk | No concerns | Major concerns | No concerns | No concerns | Very low | [] |
| OP-OCS:TT | 0 | Some concerns | Low risk | No concerns | Major concerns | No concerns | No concerns | Very low | [] |
| OP-OCS:TT-ILL | 0 | Some concerns | Low risk | No concerns | Major concerns | No concerns | No concerns | Very low | [] |

Supplementary Table26.GRADE certainty of evidence for the Marked Effective Rate outcome in IH

| **Comparison** | **Number of studies** | **Within-study bias** | **Reporting bias** | **Indirectness** | **Imprecision** | **Heterogeneity** | **Incoherence** | **Confidence rating** | **Reason**  **(s) for downgrading** |
| --- | --- | --- | --- | --- | --- | --- | --- | --- | --- |
| ILP:OP | 2 | Some concerns | Low risk | No concerns | Some concerns | Some concerns | No concerns | Very low | [] |
| ILP:TP | 1 | Some concerns | Low risk | No concerns | Major concerns | No concerns | No concerns | Very low | [] |
| Laser:Laser-TP | 1 | Some concerns | Low risk | No concerns | No concerns | Major concerns | No concerns | Very low | [] |
| Laser:OP-Laser | 2 | Some concerns | Low risk | No concerns | No concerns | Major concerns | No concerns | Very low | [] |
| Laser:TT | 1 | Some concerns | Low risk | No concerns | Major concerns | No concerns | No concerns | Very low | [] |
| OP-OCS:OP | 1 | Some concerns | Low risk | No concerns | Major concerns | No concerns | No concerns | Very low | [] |
| OP:OP-ILL | 1 | No concerns | Low risk | No concerns | Major concerns | No concerns | No concerns | Low | [] |
| OP:OP-Laser | 1 | Some concerns | Low risk | No concerns | Some concerns | No concerns | No concerns | Low | [] |
| OP:TP | 1 | Some concerns | Low risk | No concerns | Major concerns | No concerns | No concerns | Very low | [] |
| OP:TT | 2 | Some concerns | Low risk | No concerns | Major concerns | No concerns | No concerns | Very low | [] |
| OP:TT-OP | 1 | Some concerns | Low risk | No concerns | Major concerns | No concerns | No concerns | Very low | [] |
| TT:TT-ILL | 1 | Some concerns | Low risk | No concerns | Major concerns | No concerns | No concerns | Very low | [] |
| TT:TT-OP | 1 | Some concerns | Low risk | No concerns | Major concerns | No concerns | No concerns | Very low | [] |
| ILP:Laser | 0 | Some concerns | Low risk | No concerns | Major concerns | No concerns | No concerns | Very low | [] |
| ILP:Laser-TP | 0 | Some concerns | Low risk | No concerns | No concerns | Major concerns | No concerns | Very low | [] |
| ILP: OP-OCS | 0 | Some concerns | Low risk | No concerns | Major concerns | No concerns | No concerns | Very low | [] |
| ILP:OP-ILL | 0 | Some concerns | Low risk | No concerns | Major concerns | No concerns | No concerns | Very low | [] |
| ILP:OP-Laser | 0 | Some concerns | Low risk | No concerns | No concerns | Major concerns | No concerns | Very low | [] |
| ILP:TT | 0 | Some concerns | Low risk | No concerns | Some concerns | No concerns | No concerns | Low | [] |
| ILP:TT-ILL | 0 | Some concerns | Low risk | No concerns | No concerns | Major concerns | No concerns | Very low | [] |
| ILP:TT-OP | 0 | Some concerns | Low risk | No concerns | No concerns | Major concerns | No concerns | Very low | [] |
| Laser: OP-OCS | 0 | Some concerns | Low risk | No concerns | Major concerns | No concerns | No concerns | Very low | [] |
| Laser:OP | 0 | Some concerns | Low risk | No concerns | Major concerns | No concerns | No concerns | Very low | [] |
| Laser:OP-ILL | 0 | Some concerns | Low risk | No concerns | Major concerns | No concerns | No concerns | Very low | [] |
| Laser:TP | 0 | Some concerns | Low risk | No concerns | Major concerns | No concerns | No concerns | Very low | [] |
| Laser:TT-ILL | 0 | Some concerns | Low risk | No concerns | Major concerns | No concerns | No concerns | Very low | [] |
| Laser:TT-OP | 0 | Some concerns | Low risk | No concerns | Major concerns | No concerns | No concerns | Very low | [] |
| Laser-TP: OP-OCS | 0 | Some concerns | Low risk | No concerns | Major concerns | No concerns | No concerns | Very low | [] |
| Laser-TP:OP | 0 | Some concerns | Low risk | No concerns | Some concerns | No concerns | No concerns | Low | [] |
| Laser-TP:OP-ILL | 0 | Some concerns | Low risk | No concerns | Major concerns | No concerns | No concerns | Very low | [] |
| Laser-TP:OP-Laser | 0 | Some concerns | Low risk | No concerns | Major concerns | No concerns | No concerns | Very low | [] |
| Laser-TP:TP | 0 | Some concerns | Low risk | No concerns | No concerns | Major concerns | No concerns | Very low | [] |
| Laser-TP:TT | 0 | Some concerns | Low risk | No concerns | Major concerns | No concerns | No concerns | Very low | [] |
| Laser-TP:TT-ILL | 0 | Some concerns | Low risk | No concerns | Major concerns | No concerns | No concerns | Very low | [] |
| Laser-TP:TT-OP | 0 | Some concerns | Low risk | No concerns | Major concerns | No concerns | No concerns | Very low | [] |
| OP-OCS:OP-ILL | 0 | No concerns | Low risk | No concerns | Major concerns | No concerns | No concerns | Low | [] |
| OP-OCS:OP-Laser | 0 | Some concerns | Low risk | No concerns | Major concerns | No concerns | No concerns | Very low | [] |
| OP-OCS:TP | 0 | Some concerns | Low risk | No concerns | Major concerns | No concerns | No concerns | Very low | [] |
| OP-OCS:TT | 0 | Some concerns | Low risk | No concerns | Major concerns | No concerns | No concerns | Very low | [] |
| OP-OCS:TT-ILL | 0 | Some concerns | Low risk | No concerns | Major concerns | No concerns | No concerns | Very low | [] |
| OP-OCS:TT-OP | 0 | Some concerns | Low risk | No concerns | Major concerns | No concerns | No concerns | Very low | [] |
| OP:TT-ILL | 0 | Some concerns | Low risk | No concerns | Major concerns | No concerns | No concerns | Very low | [] |
| OP-ILL:OP-Laser | 0 | Some concerns | Low risk | No concerns | Major concerns | No concerns | No concerns | Very low | [] |
| OP-ILL:TP | 0 | Some concerns | Low risk | No concerns | Major concerns | No concerns | No concerns | Very low | [] |
| OP-ILL:TT | 0 | Some concerns | Low risk | No concerns | Major concerns | No concerns | No concerns | Very low | [] |
| OP-ILL:TT-ILL | 0 | Some concerns | Low risk | No concerns | Major concerns | No concerns | No concerns | Very low | [] |
| OP-ILL:TT-OP | 0 | Some concerns | Low risk | No concerns | Major concerns | No concerns | No concerns | Very low | [] |
| OP-Laser:TP | 0 | Some concerns | Low risk | No concerns | Major concerns | No concerns | No concerns | Very low | [] |
| OP-Laser:TT | 0 | Some concerns | Low risk | No concerns | Major concerns | No concerns | No concerns | Very low | [] |
| OP-Laser:TT-ILL | 0 | Some concerns | Low risk | No concerns | Major concerns | No concerns | No concerns | Very low | [] |
| OP-Laser:TT-OP | 0 | Some concerns | Low risk | No concerns | Major concerns | No concerns | No concerns | Very low | [] |
| TP:TT | 0 | Some concerns | Low risk | No concerns | Major concerns | No concerns | No concerns | Very low | [] |
| TP:TT-ILL | 0 | Some concerns | Low risk | No concerns | Major concerns | No concerns | No concerns | Very low | [] |
| TP:TT-OP | 0 | Some concerns | Low risk | No concerns | Major concerns | No concerns | No concerns | Very low | [] |
| TT-ILL:TT-OP | 0 | Some concerns | Low risk | No concerns | Major concerns | No concerns | No concerns | Very low | [] |

Supplementary Table27.GRADE certainty of evidence for the Total Effective Rate outcome in IH

| **Comparison** | **Number of studies** | **Within-study bias** | **Reporting bias** | **Indirectness** | **Imprecision** | **Heterogeneity** | **Incoherence** | **Confidence rating** | **Reason**  **(s) for downgrading** |
| --- | --- | --- | --- | --- | --- | --- | --- | --- | --- |
| Laser:Laser-TP | 1 | Some concerns | Low risk | No concerns | Some concerns | No concerns | No concerns | Low | [] |
| Laser:OP-Laser | 2 | Some concerns | Low risk | No concerns | No concerns | No concerns | No concerns | Moderate | [] |
| Laser:TT | 1 | Some concerns | Low risk | No concerns | Major concerns | No concerns | No concerns | Very low | [] |
| OP:OP-Laser | 1 | Some concerns | Low risk | No concerns | No concerns | No concerns | No concerns | Moderate | [] |
| TT:TT-ILL | 1 | Some concerns | Low risk | No concerns | No concerns | No concerns | No concerns | Moderate | [] |
| Laser:OP | 0 | Some concerns | Low risk | No concerns | Major concerns | No concerns | No concerns | Very low | [] |
| Laser:TT-ILL | 0 | Some concerns | Low risk | No concerns | Major concerns | No concerns | No concerns | Very low | [] |
| Laser-TP:OP | 0 | Some concerns | Low risk | No concerns | Major concerns | No concerns | No concerns | Very low | [] |
| Laser-TP:OP-Laser | 0 | Some concerns | Low risk | No concerns | Major concerns | No concerns | No concerns | Very low | [] |
| Laser-TP:TT | 0 | Some concerns | Low risk | No concerns | Major concerns | No concerns | No concerns | Very low | [] |
| Laser-TP:TT-ILL | 0 | Some concerns | Low risk | No concerns | Major concerns | No concerns | No concerns | Very low | [] |
| OP:TT | 0 | Some concerns | Low risk | No concerns | Major concerns | No concerns | No concerns | Very low | [] |
| OP:TT-ILL | 0 | Some concerns | Low risk | No concerns | Major concerns | No concerns | No concerns | Very low | [] |
| OP-Laser:TT | 0 | Some concerns | Low risk | No concerns | Major concerns | No concerns | No concerns | Very low | [] |
| OP-Laser:TT-ILL | 0 | Some concerns | Low risk | No concerns | Major concerns | No concerns | No concerns | Very low | [] |

Supplementary Table28.GRADE certainty of evidence for the TEAE outcome in IH

| **Comparison** | **Number of studies** | **Within-study bias** | **Reporting bias** | **Indirectness** | **Imprecision** | **Heterogeneity** | **Incoherence** | **Confidence rating** | **Reason**  **(s) for downgrading** |
| --- | --- | --- | --- | --- | --- | --- | --- | --- | --- |
| Laser:OP-Laser | 2 | Some concerns | Low risk | No concerns | Some concerns | Some concerns | Some concerns | Very low | [] |
| Laser:TT | 1 | Some concerns | Low risk | No concerns | Some concerns | No concerns | Some concerns | Very low | [] |
| OA:OP | 2 | No concerns | Low risk | No concerns | Some concerns | Some concerns | Some concerns | Very low | [] |
| OCS:OP | 2 | No concerns | Low risk | No concerns | Some concerns | No concerns | Some concerns | Low | [] |
| ON:OP | 1 | No concerns | Low risk | No concerns | Some concerns | No concerns | Some concerns | Low | [] |
| OP:OP-Laser | 1 | Some concerns | Low risk | No concerns | Some concerns | Some concerns | Some concerns | Very low | [] |
| OP:TP | 1 | Some concerns | Low risk | No concerns | Some concerns | No concerns | Some concerns | Very low | [] |
| WW:TT | 1 | Some concerns | Low risk | No concerns | Some concerns | Some concerns | Some concerns | Very low | [] |
| Laser:OA | 0 | Some concerns | Low risk | No concerns | Some concerns | No concerns | Some concerns | Very low | [] |
| Laser:OCS | 0 | Some concerns | Low risk | No concerns | Some concerns | No concerns | Some concerns | Very low | [] |
| Laser:ON | 0 | Some concerns | Low risk | No concerns | Some concerns | No concerns | Some concerns | Very low | [] |
| Laser:OP | 0 | Some concerns | Low risk | No concerns | Some concerns | No concerns | Some concerns | Very low | [] |
| Laser:WW | 0 | Some concerns | Low risk | No concerns | Some concerns | No concerns | Some concerns | Very low | [] |
| Laser:TP | 0 | Some concerns | Low risk | No concerns | Some concerns | No concerns | Some concerns | Very low | [] |
| OA:OCS | 0 | No concerns | Low risk | No concerns | Some concerns | No concerns | Some concerns | Low | [] |
| OA:ON | 0 | No concerns | Low risk | No concerns | Some concerns | No concerns | Some concerns | Low | [] |
| OA:OP-Laser | 0 | Some concerns | Low risk | No concerns | Some concerns | No concerns | Some concerns | Very low | [] |
| OA:WW | 0 | Some concerns | Low risk | No concerns | Some concerns | No concerns | Some concerns | Very low | [] |
| OA:TP | 0 | Some concerns | Low risk | No concerns | Some concerns | No concerns | Some concerns | Very low | [] |
| OA:TT | 0 | Some concerns | Low risk | No concerns | Some concerns | No concerns | Some concerns | Very low | [] |
| OCS:ON | 0 | No concerns | Low risk | No concerns | Some concerns | No concerns | Some concerns | Low | [] |
| OCS:OP-Laser | 0 | Some concerns | Low risk | No concerns | Some concerns | Some concerns | Some concerns | Very low | [] |
| OCS:WW | 0 | Some concerns | Low risk | No concerns | Some concerns | No concerns | Some concerns | Very low | [] |
| OCS:TP | 0 | Some concerns | Low risk | No concerns | Some concerns | No concerns | Some concerns | Very low | [] |
| OCS:TT | 0 | Some concerns | Low risk | No concerns | Some concerns | No concerns | Some concerns | Very low | [] |
| ON:OP-Laser | 0 | No concerns | Low risk | No concerns | Some concerns | No concerns | Some concerns | Low | [] |
| ON:WW | 0 | Some concerns | Low risk | No concerns | Some concerns | No concerns | Some concerns | Very low | [] |
| ON:TP | 0 | No concerns | Low risk | No concerns | Some concerns | No concerns | Some concerns | Low | [] |
| ON:TT | 0 | Some concerns | Low risk | No concerns | Some concerns | No concerns | Some concerns | Very low | [] |
| OP:WW | 0 | Some concerns | Low risk | No concerns | Some concerns | No concerns | Some concerns | Very low | [] |
| OP:TT | 0 | Some concerns | Low risk | No concerns | Some concerns | No concerns | Some concerns | Very low | [] |
| OP-Laser:WW | 0 | Some concerns | Low risk | No concerns | Some concerns | No concerns | Some concerns | Very low | [] |
| OP-Laser:TP | 0 | Some concerns | Low risk | No concerns | Some concerns | No concerns | Some concerns | Very low | [] |
| OP-Laser:TT | 0 | Some concerns | Low risk | No concerns | Some concerns | No concerns | Some concerns | Very low | [] |
| WW:TP | 0 | Some concerns | Low risk | No concerns | Some concerns | No concerns | Some concerns | Very low | [] |
| TP:TT | 0 | Some concerns | Low risk | No concerns | Some concerns | No concerns | Some concerns | Very low | [] |

Supplementary Table29.GRADE certainty of evidence for the Recurrence Rate outcome in IH

| **Comparison** | **Number of studies** | **Within-study bias** | **Reporting bias** | **Indirectness** | **Imprecision** | **Heterogeneity** | **Incoherence** | **Confidence rating** | **Reason**  **(s) for downgrading** |
| --- | --- | --- | --- | --- | --- | --- | --- | --- | --- |
| ILP:OP | 1 | Some concerns | Low risk | No concerns | Some concerns | No concerns | No concerns | Low | [] |
| ILP:TP | 1 | Some concerns | Low risk | No concerns | Some concerns | No concerns | No concerns | Low | [] |
| OA:OP | 1 | No concerns | Low risk | No concerns | Some concerns | Low risk | No concerns | Moderate | [] |
| OP-OCS:OP | 1 | Some concerns | Low risk | No concerns | Some concerns | No concerns | No concerns | Low | [] |
| OP:TP | 2 | Some concerns | Low risk | No concerns | Some concerns | No concerns | No concerns | Low | [] |
| ILP:OA | 0 | Some concerns | Low risk | No concerns | Some concerns | No concerns | No concerns | Low | [] |
| ILP: OP-OCS | 0 | Some concerns | Low risk | No concerns | Some concerns | No concerns | No concerns | Low | [] |
| OA: OP-OCS | 0 | No concerns | Low risk | No concerns | Some concerns | Low risk | No concerns | Moderate | [] |
| OA:TP | 0 | Some concerns | Low risk | No concerns | Some concerns | No concerns | No concerns | Low | [] |
| OP-OCS:TP | 0 | Some concerns | Low risk | No concerns | Some concerns | No concerns | No concerns | Low | [] |

Supplementary Table30.GRADE certainty of evidence for the Marked Effective Rate outcome in superficial IH

| **Comparison** | **Number of studies** | **Within-study bias** | **Reporting bias** | **Indirectness** | **Imprecision** | **Heterogeneity** | **Incoherence** | **Confidence rating** | **Reason**  **(s) for downgrading** |
| --- | --- | --- | --- | --- | --- | --- | --- | --- | --- |
| Laser:OP-Laser | 1 | Some concerns | Low risk | No concerns | Some concerns | No concerns | No concerns | Low | [] |
| OP:OP-Laser | 1 | Some concerns | Low risk | No concerns | No concerns | No concerns | No concerns | Moderate | [] |
| OP:TT | 2 | Some concerns | Low risk | No concerns | Major concerns | No concerns | No concerns | Very low | [] |
| OP:TT-OP | 1 | Some concerns | Low risk | No concerns | Some concerns | No concerns | No concerns | Low | [] |
| TT:TT-OP | 1 | Some concerns | Low risk | No concerns | Major concerns | No concerns | No concerns | Very low | [] |
| Laser:OP | 0 | Some concerns | Low risk | No concerns | Major concerns | No concerns | No concerns | Very low | [] |
| Laser:TT | 0 | Some concerns | Low risk | No concerns | Major concerns | No concerns | No concerns | Very low | [] |
| Laser:TT-OP | 0 | Some concerns | Low risk | No concerns | Major concerns | No concerns | No concerns | Very low | [] |
| OP-Laser:TT | 0 | Some concerns | Low risk | No concerns | No concerns | Major concerns | No concerns | Very low | [] |
| OP-Laser:TT-OP | 0 | Some concerns | Low risk | No concerns | Major concerns | No concerns | No concerns | Very low | [] |

Supplementary Figure3. SUCRA ranking plot table for the Excellent Response Rate outcome in IH

Supplementary Figure4. SUCRA ranking plot table for the Marked Effective Rate outcome in IH

Supplementary Figure5. SUCRA ranking plot table for the Total Effective Rate outcome in IH

Supplementary Figure6. SUCRA ranking plot table for the VAS (Color) outcome in IH

Supplementary Figure7. SUCRA ranking plot table for the VAS (Size) outcome in IH

Supplementary Figure8. SUCRA ranking plot table for the HAS outcome in IH

Supplementary Figure9. SUCRA ranking plot table for the Healing time outcome in IH

Supplementary Figure10. SUCRA ranking plot table for the TEAE outcome in IH

Supplementary Figure11. SUCRA ranking plot table for the Recurrence Rate outcome in IH

Supplementary Figure12. SUCRA ranking plot for the Marked Effective Rate outcome in Superficial IH

Supplementary Figure13. Sensitivity analysis of the network meta-analysis for the HAS outcome using alternative correlation coefficients (R=0.25 and R=0.75)

Supplementary Table31. Original definitions and assessment criteria of treatment response across included trials.

| Study | Original definition of treatment response |
| --- | --- |
| Gong et al.26 | Graded on a 4-point scale as proposed by Achauer et al: class I (poor) - reduction in size of <25%; class II (moderate) - reduction in size of 25% - 50%; class III (good) - reduction in size of 50% - 75%; and class IV (excellent) - reduction in size of 75% - 100%. |
| Zhao et al. 29 | Treatment efficacy was evaluated using the internationally accepted four-level grading system: Grade I, 0–25% reduction in tumor size; Grade II, 26–50% reduction; Grade III, 51–75% reduction; and Grade IV, 76–100% reduction. |
| Ehsani et al.32 | Clinical responses to the treatment regimens were evaluated with respect to the cessation of the growth, decreases in the size, and lightening of the color of lesions. The visual scores were classified into four categories as follows: score 1, 0–25% improvement (no response); score 2, 25–50% improvement (poor response); score 3, 50–75% improvement (good response); and score 4, 75–100% improvement (excellent response). |
| Liu et al.33 | The efficacy depends on the grading standards of Achauer, the disease proved to be cured when the tumor area reduced to a range of 76%-100%; the efficacy proved to be marked when the tumor area reduced to a range of 51%-75%; the efficacy proved to be effective when the tumor area reduced to a range of 26%-50%; the efficacy proved to be invalid when the tumor area reduced to a range within 25%; total efficacy=cured + marked + effective. |
| Sun et al.34 | Cured: the tumor body in the infant patients was decreased by ≥76.0% after treatment. Markedly effective: the tumor body in the infant patients was decreased by 51.0‑75.0% after treatment. Effective: the tumor body in the infant patients was decreased by 26.0‑50.0% after treatment. Ineffective: the tumor body in the infant patients was decreased by <25.0% after treatment |
| Yang et al.35 | Ineffective, Grade I, tumor shrinkage ≤25%; improved, Grade II, tumor shrinkage >25% and ≤50%; basically cured, Grade III, tumor shrinkage >50% and ≤75%, with skin color close to normal or mild pigmentation, functional recovery, but incomplete recovery of appearance; cured, Grade IV, tumor shrinkage >75%, normal skin color, functional recovery, and no recurrence during follow-up. The total effective rate was calculated as: Total effective rate = (total number of cases − number of Grade I cases) / total number of cases × 100%. |
| Zaher et al.36 | Excellent (complete resolution achieved), good (sustained plateau, with≥50% reduction in size of IH), fair (sustained plateau, with <50% reduction in size of IH) or poor (no response, worsening of IH, in tolerable side effects necessitating stopping therapy) |
| Tawfik et al.37 | 0, no improvement; <25%, mild improvement; 26% to 50%, moderate improvement; 51% to 75%, good improvement; 76% to 100%, excellent improvement |
| Malik et al.40 | Measure of assessment for color and size was based on Visual Analogue Scale (VAS) by comparing follow-up images to the baseline photograph pretreatment. The images were evaluated by two independent blinded examiners who scored the improvement as: 0–24%, 25%–49%, 50%–74% and 75%–100%. |
| Mehta et al.45 | Efficacy of therapy was measured as regression in size on clinical examination. This was further categorized into a scale as excellent (≥90%), very good (70 to 89.9%), good (50–69.9%), fair (30–49.9%), and poor (<30%) |
| Aly et al.18 | The response to the treatment was evaluated based on size reduction according to Pandey et al.. Response was defined as excellent with more than 75 % reduction in size, moderate with 50–75 % reduction, mild with 25–49 % reduction, poor less than 25 % reduction, while no response was considered with 0 % reduction. |
| Guo et al.46 | Tumor dimensions were measured by ultrasound to calculate volume (Volume = length ×width ×height ×0.52). Therapeutic effect was categorized as: Complete Regression (>95% volume reduction), Marked Effectiveness(≥75%-95%), Moderate Effectiveness (30%to<75%), or Ineffective (<30%). |
| Ma et al.47 | Grade 1 (excellent final response), means complete resolution achieved; Grade 2 (good final response), means with ≥50% reduction in size of IH; Grade 3 (fair final response), means with <50% reduction in size of IH; Grade 4 (poor final response), no response or worsening of IH. |
| Zhang et al.48 | Treatment efficacy was evaluated using the grading criteria proposed by Achauer et al. Grade I was defined as tumor shrinkage ≤25% or partial fading of the surface color compared with before treatment; Grade II was defined as tumor shrinkage >25% and ≤50% or obvious fading of the surface color; Grade III was defined as tumor shrinkage >50% and ≤75% with obvious fading of the surface color; and Grade IV was defined as tumor shrinkage >75% or complete fading of the surface color. The total effective rate was calculated as: Total effective rate = (number of Grade III cases + number of Grade IV cases) / total number of cases. |

Supplementary Table32. Summary of baseline clinical characteristics and study parameters across different treatment comparisons.

| Study | Treatment comparison | treatment indication | Anatomical location | Disease stage | Baseline lesion burden | Baseline severity scale / activity score | High-risk features or complications | Previous IH treatment | Follow-up duration |
| --- | --- | --- | --- | --- | --- | --- | --- | --- | --- |
| Gong et al.26 | Topical timolol + oral propranolol vs oral propranolol vs topical timolol | superficial IH | eyelids, lips, nose, ears, parotid, cheek | NR | NR | NR | Potential disfigurement or functional threat | No previous treatment required | 3–12 months |
| Ji et al.27 | Oral atenolol vs oral propranolol | Infantile hemangioma | Head, face, neck, trunk, extremity | Proliferating IH | Atenolol arm: lesion size 8.8 cm², median IQR 5.0–18.0; Propranolol arm: lesion size 8.1 cm², median IQR 5.5–15.3; | HAS at baseline: atenolol 4.54±0.76; propranolol 4.61±0.82. | ulceration: atenolol 23/187, propranolol 25/19S0. | Previous IH treatment excluded | At least 6 months of treatment; |
| Ashraf et al.28 | Oral propranolol vs oral atenolol | Problematic IH, including complicated and/or cosmetically significant IH; | Head, neck, trunk, extremity | Mostly proliferative phase | Propranolol arm: baseline USG volume 3.48 ± 3.19 cm³, range 1.15–12.48, Atenolol arm: baseline USG volume 3.2 ± 4.86 cm³, range 0.29–14.3 | HAS at baseline: propranolol 4.55±0.99; atenolol 4.92±0.83 | complicated/cosmetically significant IH. | NR | 9 months |
| Zhao et al. 29 | Topical timolol vs oral propranolol | Superficial infantile hemangioma | Face, neck, trunk, scalp, perineum limbs | NR | Overall lesion size ranged from 0.5 × 0.2 cm to 5.0 × 5.0 cm | NR | NR | NR | 4-13 months |
| Li et al.30 | Oral propranolol + topical timolol maleate vs oral propranolol | Mixed infantile hemangiomas in the oral and maxillofacial regions | lip, cheek, parotid area, temple. nose, eyelid, forehead, periocular | NR | Overall lesion size ranged from 1.0 × 1.0 cm to 5.5 × 6.0 cm | VAS was used to assess treatment response in size and color ranged from 4 to 10 | Functional or cosmetic deformity. Lesions were located in oral/maxillofacial sites | Previous IH treatment excluded | First follow-up after 2 weeks, then monthly |
| Marey et al.31 | Oral propranolol + topical timolol vs oral propranolol | Superficial periocular infantile capillary hemangioma with functional visual impairment | Periocular / eyelid region | Early proliferative stage | NR | HAS at baseline: propranolol + timolol 4.59 ± 1.04; propranolol 4.80 ± 0.71 | Functional visual impairment | No previous treatment required | Patients were followed every 4 weeks. |
| Ehsani et al.32 | PDL + topical propranolol vs PDL | Superficial and mixed infantile hemangiomas; lesions located on non-vital body sites | head/face, limbs, trunk, genitalia | NR | NR | NR | One ulcerated lesion was present in each group | No previous systemic steroids or other treatment modalities within the previous 3 months | 4 months |
| Liu et al.33 | PDL + oral propranolol vs PDL | Superficial hemangioma in infants/children | NR | NR | NR | NR | NR | NR | Exact total follow-up not clearly reported. |
| Sun et al.34 | Oral propranolol + laser vs laser | single hemangioma | NR | NR | NR | NR | NR | NR | 3-6 months |
| Yang et al.35 | Topical propranolol + PDL vs topical propranolol | Superficial infantile hemangioma | Head, neck, trunk, limbs | NR | Topical propranolol + PDL baseline tumor area 2.07 ± 0.16 cm²; topical propranolol baseline tumor area 2.12 ± 0.13 cm² | NR | NR | Prior laser treatment was excluded | 6 months |
| Zaher et al.36 | Topical propranolol vs intralesional propranolol vs oral propranolol | Problematic IH | Head, neck, buttocks, trunk, limbs | NR | Topical propranolol: lesion size 3–10 cm², mean 6 cm²; intralesional propranolol: lesion size 4–15 cm², mean 7.67 cm², oral propranolol: lesion size 4–15 cm², mean 8 cm². | NR | Rapidly progressive IH, IH compromising vital functions, affecting normal physiological functions, causing cosmetic disfigurement | Any previous IH treatment was discontinued for 3 months before inclusion | Patients were followed weekly during the first month, every 2 weeks during the second month, and then every 4 weeks until 6 months after treatment cessation |
| Tawfik et al.37 | Topical timolol vs 595 nm PDL + 1,064 nm Nd:YAG | superficial and mixed IH | Head, trunk, vulva, extremities | NR | Topical timolol: lesion size 0.5–2.0 cm² 13/30, >2–10 cm² 8/30, >10 cm² 9/30; Laser arm, n=30: lesion size 0.5–2.0 cm² 14/30, >2–10 cm² 11/30, >10 cm² 5/30; | Objective redness / vascularity baseline assessed by Antera 3D average hemoglobin level: timolol 3.18 ± 0.92, laser 3.76 ± 1.03 | NR | Previous treatment was excluded | 3 months after the last treatment session |
| Bauman et al.38 | Oral propranolol vs oral prednisolone | Symptomatic infantile hemangiomas | Periorbital, nasal tip, ear, lip, beard area, frontotemporal, breast, forehead, cheek | Actively proliferating IH | Propranolol: baseline TSA median 5.0 cm², 95% CI 1.9–25.6; baseline AdjTSA median 5.0 cm², 95% CI 1.5–24.3; prednisolone: baseline TSA median 3.1 cm², 95% CI 0.8–10.5; baseline AdjTSA median 2.5 cm², 95% CI 0.2–9.4. | NR | Impaired function, including breathing, eating, vision or hearing, were ulcerated, caused pain, or were located in cosmetically sensitive regions | Other treatment for IH was excluded | 4 months |
| Kim et al.39 | Oral propranolol vs oral prednisolone | At least one of the following: 10%–20% volume increase within 2–4 weeks, IH-related organ dysfunction, or IH-related aesthetic problem | Scalp, face, chest, abdomen, back, extremity | NR | propranolol: baseline MRI volume 14,125.35 ± 18,246.77 mm³; surface area 1,318.06 ± 1,833.07 mm²; height 4.26 ± 2.22 mm. Ulceration 1.  prednisolone: baseline MRI volume 9,349.54 ± 16,015.69 mm³; surface area 1,093.51 ± 1,316.68 mm²; height 3.71 ± 2.49 mm. Ulceration 1 | NR | Treatment indication included rapidly increasing lesion volume, organ dysfunction, or aesthetic problem | Previous IH treatment was excluded | 5 months |
| Malik et al.40 | Oral propranolol vs oral prednisolone vs oral propranolol + prednisolone | Potentially disfiguring or functionally threatening infantile hemangiomas | head and neck, lip, scalp | Proliferative / early treatment phase implied | NR | NR | Problematic IH, included facial disfiguring lesions and functionally threatening lesions involving limbs, genitalia, or natural orifices | No patient was on concomitant therapy at treatment initiation | 11 months |
| Tan et al.41 | Oral propranolol + 1064 nm Nd:YAG laser vs 1064 nm Nd:YAG laser vs oral propranolol | Body-surface infantile hemangioma requiring active treatment | Body-surface | Proliferating hemangioma | with longest transverse diameter >1.0 cm | NR | NR | All cases had not received previous treatment before enrollment | 6 months |
| Abdel Wahab et al.42 | Oral propranolol vs topical propranolol | Superficial cutaneous infantile hemangioma | head/neck, trunk, extremities | NR | Oral propranolol: lesion size 0.5–2 cm 12, 2–10 cm 8, >10 cm 4; single lesion 22, multiple lesions 2; Topical propranolol: lesion size 0.5–2 cm 15, 2–10 cm 5, >10 cm 4; single lesion 21, multiple lesions 3 | NR | Functional impairment, local discomfort, or esthetic disfigurement | Previous treatment was excluded | 9 months |
| Pope et al.43 | Oral nadolol vs oral propranolol | problematic infantile hemangioma | Head, neck | NR | NR | Baseline HSS: nadolol 10.6 ± 3.4, propranolol 10.61 ± 3.3. Baseline HAS: nadolol 8.59 ± 1.8, propranolol 9.58 ± 1.4 | Complications: visual obstruction, feeding problems, ulceration, distortion of landmarks, nasal obstruction. | Patients receiving oral β-blocker or other systemic agents within 2 weeks and receiving topical timolol within 1 week were excluded | 12 months |
| Cheng et al.44 | Topical timolol vs watchful waiting | mall superficial IH located in high-risk areas | face, scalp, acral, neck, buttocks | NR | Small superficial IH <2 cm in longest diameter. Timolol: Baseline IH volume 153.2 ± 184.1 mm³; watchful waiting: Baseline IH volume 99.44 ± 120.0 mm³ | NR | All lesions were in predefined high-risk areas: tip of ears, tip of nose, eyelids, acral areas, facial areas, scalp, neck, buttocks, perineum, or axilla | Pre-treated IH were excluded. | 12 months |
| Mehta et al.45 | ntralesional propranolol vs oral propranolol | Periorbital and eyelid capillary hemangiomas requiring treatment | Periorbital and eyelid region | NR | intralesional propranolol: baseline cross-sectional area 332.3 ± 261.94 mm², Baseline astigmatism 1.617 D at 90.16°; oral propranolol: baseline cross-sectional area 615.77 ± 592.67 mm². Baseline astigmatism 2.8588 D at 179.8° | NR | Periorbital/eyelid location itself represents a high-risk anatomical site because of risk of ptosis, astigmatism, amblyopia, exposure keratopathy, optic nerve compression, and visual-axis obstruction | Previously treated lesions were excluded | 6 months |
| Aly et al.18 | Oral propranolol + oral prednisolone vs oral propranolol alone | Cutaneous infantile hemangioma causing complications | Face, neck, limb, anterior abdominal wall | NR | propranolol + prednisolone: volume 60 cm³, IQR 19.3–82.5; propranolol: volume 14.5 cm³, IQR 4.8–47.3 | NR | complicated IH | Previous treatment for IH was excluded | 9 months |
| Guo et al.46 | Oral propranolol + intralesional bleomycin vs oral propranolol | Infantile hemangioma requiring treatment | Head, neck, trunk, limb | NR | NR | NR | high-risk location, functional impairment risk, ulceration, or cosmetic concern | No prior IH treatment was allowed | 6 months |
| Ma et al.47 | Oral propranolol + intralesional lauromacrogol vs oral propranolol | the lesion affected appearance or health | Head, face, neck, limb, trunk, mixed location | NR | propranolol + lauromacrogol: baseline lesion diameter 2.7 ± 1.1 cm; propranolol： baseline lesion diameter 3.1 ± 1.9 cm | NR | lesions on face, trunk, or limbs that affected appearance or health | without any previous treatment | 6 monrhs |
| Zhang et al.48 | Topical timolol + lauromacrogol injection vs topical timolol | Infantile hemangioma | Head, face, neck, trunk, limbs | NR | Timolol + lauromacrogol: tumor diameter 2.03 ± 0.27 cm; baseline hemangioma thickness 4.12 ± 0.48 mm; Timolol: tumor diameter 2.11 ± 0.39 cm; baseline hemangioma thickness 4.06 ± 0.46 mm | Baseline size VAS: Timolol + lauromacrogol: 1.13 ± 0.62, Timolol 1.15 ± 0.61. Baseline color VAS: Timolol + lauromacrogol 2.18 ± 0.42, Timolol 2.16 ± 0.43. | NR | Previous treatment after disease onset was excluded | 6 months |
| Kashif et al.49 | Oral propranolol vs oral steroid | Infantile hemangioma requiring drug treatment | Scalp, face, chest, abdomen, back, extremity | NR | Propranolol: baseline MRI volume 29,672.70 ± 25.17 mm³; baseline surface area 4,099.01 ± 49.72 mm²; baseline height 8.40 ± 3.12 mm; steroid: baseline MRI volume 29,682.8 ± 24.31 mm³; baseline surface area 4,145.34 ± 44.19 mm²; baseline height 9.87 ± 2.55 mm. | NR | NR | Previous IH treatment was excluded | 5 months |

Supplementary Table33. Definitions, intervention categories, and clinical parameters of treatment nodes included in the network meta-analysis.

| Treatment node in NMA | Full name | Intervention category | Definition of the node | Dose, intensity, or treatment parameters |
| --- | --- | --- | --- | --- |
| OP | Oral propranolol | Oral systemic β-blocker | Oral propranolol monotherapy | 0.5 mg/kg/d for 2 week, increased to 0.8 mg/kg/d in week 3, increased to 1 mg/kg/d in week 5;  1.0 mg/kg/d;  1.5 mg/kg/d;  1 mg/kg/d for 1 week and increased to 2.0mg/kg/d in week 2;  1 mg/kg/d for 2 week and increased to 2.0mg/kg/d in week 3;  2.0 mg/kg/d;  3.0 mg/kg/d; |
| OA | Oral atenolol | Oral systemic β-blocker | Oral atenolol monotherapy | 1 mg/kg/d;  0.5 mg/kg/d for 1 week and increased to 1.0mg/kg/d in week 2; |
| ON | Oral nadolol | Oral systemic β-blocker | Oral nadolol monotherapy | 2mg/kg/d |
| OCS | Oral prednisolone | Oral systemic corticosteroid | Oral prednisolone monotherapy | 1.0 mg/kg/d  2.0 mg/kg/d; |
| TT | Topical timolol | Topical β-blocker | Topical timolol monotherapy | 0.5% timolol once daily;  0.5% timolol twice daily; |
| TP | Topical propranolol | Topical β-blocker | Topical propranolol monotherapy | propranolol 1%, twice daily;  propranolol 3 times a day; |
| Laser | Laser therapy | Local procedural therapy | Laser monotherapy, including PDL, Nd:YAG, | **PDL:**  spot size 7 mm, 12 J/cm2, pulse duration 1.5 ms, dynamic cooling device 40/40, 3 sessions, once every 4 weeks, with 50% over lapped shuts;  spot size 7 mm, 7-11 J/cm2, pulse duration 2 ms, 4-6 sessions, once every 4 weeks, with 10-20% over lapped shuts;  spot size 7 mm, 4.5-6 J/cm2, pulse duration 6 ms, 1s delay, every month for a maximum of six sessions;  spot size 7 mm, 6-7.5 J/cm2, pulse duration 10 ms, 1s delay, every month for a maximum of six sessions;  **Nd:YAG Laser:**  pulse duration 6 ms, 30-40 J/cm2, every month for a maximum of six sessions;  pulse duration 15 ms, 30-40 J/cm2, every month for a maximum of six sessions;  pulse duration 20-50 ms, 170-240 J/cm2) every 6 weeks; |
| ILP | Intralesional propranolol | Local injection β-blocker | propranolol injection monotherapy | 0.2 mL injected per 1 cm of lesion diameter with a maximum volume of 1 mL;  0.2 mL per cm of LLD with a maximum dose of 1 ml, 3 sessions, once every 4 weeks; |
| WW | Watchful waiting | Conservative management | Observation | Watchful waiting |
| OP-Laser | Oral propranolol with laser therapy | Combination therapy | Oral propranolol combined with laser therapy | PDL (spot size 7 mm, 7-11 J/cm2, pulse duration 2 ms), 4-6 sessions, once every 4 weeks, with 10-20% over lapped shuts + oral propranolol 2mg/kg/d;  long-pulse 1,064 nm Nd:YAG laser (spot size 9 mm, pulse width 12 msec, 40 70 J/cm2), every 3 weeks, with <10% over lapped + oral propranolol 2 mg/kg/d;  Nd:YAG Laser (pulse duration 20-50 ms, 170-240 J/cm2) every 6 weeks + oral propranolol 0.5 mg/kg/d for 2 week, increased to 0.8 mg/kg/d in week 3, increased to 1 mg/kg/d in week 5 |
| OP-TT | Oral propranolol with topical timolol | Combination therapy | Oral propranolol combined with topical timolol | 0.5% timolol twice daily+ oral propranolol 1.0 mg/kg/d;  0.5% timolol twice daily+ oral propranolol 1.0 mg/kg/d for 2 week and increased to 2 mg/kg/d for week 3; |
| OP-OCS | Oral propranolol with oral prednisolone | Combination therapy | Oral propranolol combined with oral prednisolone | prednisolone 1.0 mg/kg/d + propranolol 1-3 mg/kg/d;  prednisolone 2.0 mg/kg/d + propranolol 2 mg/kg/d; |
| OP-ILL | Oral propranolol with intralesional lauromacrogol | Combination therapy | Oral propranolol combined with intralesional lauromacrogol | propranolol 2.0 mg/kg/d + intralesional injection of lauromacrogol for 2-4 times within 6 months |
| Laser-TP | Laser therapy with topical propranolol | Combination therapy | Laser therapy combined with topical propranolol | PDL (spot size 7 mm, 12 J/cm2, pulse duration 1.5 ms, dynamic cooling device 40/40), 3 sessions, once every 4 weeks, with 50% over lapped shuts + topical propranolol 1%, applied twice a day;  PDL (spot size 7 mm, 8-15 J/cm2, pulse duration 0.45-10 ms, dynamic cooling device 40/40), every 2-4 weeks + topical propranolol |
| TT-ILL | Topical timolol with intralesional lauromacrogol | Combination therapy | Topical timolol combined with intralesional lauromacrogol | 0.5% timolol twice daily + intralesional injection of lauromacrogol for 1-4 times |
| OP-ILB | Oral propranolol with intralesional bleomycin | Combination therapy | Oral propranolol combined with intralesional bleomycin | propranolol 1.0 mg/kg/d + intralesional injection of bleomycin |
